# Identification of Single- and Multiple-Class Specific Signature Genes from Gene Expression Profiles by Group Marker Index

**DOI:** 10.1371/journal.pone.0024259

**Published:** 2011-09-01

**Authors:** Yu-Shuen Tsai, Kripamoy Aguan, Nikhil R. Pal, I-Fang Chung

**Affiliations:** 1 Institute of Biomedical Informatics, National Yang-Ming University, Taipei, Taiwan; 2 Department of Biotechnology & Bioinformatics, North Eastern Hill University, Shillong, India; 3 Electronics & Communication Sciences Unit, Indian Statistical Institute, Calcutta, India; 4 Center for Systems and Synthetic Biology, National Yang-Ming University, Taipei, Taiwan; Virginia Commonwealth University, United States of America

## Abstract

Informative genes from microarray data can be used to construct prediction model and investigate biological mechanisms. Differentially expressed genes, the main targets of most gene selection methods, can be classified as single- and multiple-class specific signature genes. Here, we present a novel gene selection algorithm based on a Group Marker Index (GMI), which is intuitive, of low-computational complexity, and efficient in identification of both types of genes. Most gene selection methods identify only single-class specific signature genes and cannot identify multiple-class specific signature genes easily. Our algorithm can detect *de novo* certain conditions of multiple-class specificity of a gene and makes use of a novel non-parametric indicator to assess the discrimination ability between classes. Our method is effective even when the sample size is small as well as when the class sizes are significantly different. To compare the effectiveness and robustness we formulate an intuitive template-based method and use four well-known datasets. We demonstrate that our algorithm outperforms the template-based method in difficult cases with unbalanced distribution. Moreover, the multiple-class specific genes are good biomarkers and play important roles in biological pathways. Our literature survey supports that the proposed method identifies unique multiple-class specific marker genes (not reported earlier to be related to cancer) in the Central Nervous System data. It also discovers unique biomarkers indicating the intrinsic difference between subtypes of lung cancer. We also associate the pathway information with the multiple-class specific signature genes and cross-reference to published studies. We find that the identified genes participate in the pathways directly involved in cancer development in leukemia data. Our method gives a promising way to find genes that can involve in pathways of multiple diseases and hence opens up the possibility of using an existing drug on other diseases as well as designing a single drug for multiple diseases.

## Introduction

Gene selection/biomarker identification methods can be classified as linear and nonlinear methods [1]. A linear method assumes that good biomarkers for a class will be highly expressed (or unexpressed) for that class and unexpressed (or highly expressed) for the rest of the classes [2], [3]. Nonlinear methods identify biomarkers exploiting both linear and nonlinear interactions between classes and genes [4], [5]. Most methods are linear in nature and there are a few non-linear approaches. Nonlinear approaches can discover a small set of discriminatory genes that are good enough for diagnosis of a set of diseases. But the relationship of biomarkers identified by nonlinear methods with different classes may not be easily visualized. Saeys *et al.* [6] reviewed various methods of biomarker identification and suggested three categories for the existing methodologies. The first one is the *filter* method category, which ranks genes independently of the classifier that is used. Many linear methods, either univariate or multivariate, e.g., “SAM” [7], “shrinkage *t*” [8], “correlation-adjusted *t*” [9] belong to this category. Most of these methods are essentially based on Student's t-test or its modified/adapted forms. In some cases, in conjunction with the t-test, authors have used additional procedures to account for the high-dimensional characteristic of microarray data. In addition to t-test, SAM also uses Wilcoxon's Rank Sum test. In this category, other parametric and non-parametric tests have also been used [10], [11]. The second one is the *wrapper* method category, which selects genes according to the predictive performance of the associated classifier [12]-[14]. The final category of *embedded* methods assigns weights to the importance of genes by making use of the internal parameters of the classification model – this is an integrated approach where the feature weighting/selection and classifier design are done simultaneously. The well-known SVM-RFE [4], other SVM-based methods, e.g., MMC-RFE [15], MSVM-RFE [16], SVM-RCE [17], and SVM-RNE [18] all belong to this category. Also, some neural network-based methods, e.g., online FSMLP [5], belong to the same category. The advantages of these *wrapper* and *embedded* methods are that they can capture non-linear interactions between genes as well as interaction between genes and the diseases. As a result, such marker genes usually provide a better predictive performance.

Most existing gene selection methods (particularly linear methods) can identify only single-class specific signature genes (i.e., genes that are expressed for one class and unexpressed for the rest of the classes). However, there could be genes which are expressed for a subset of classes (say, for a subgroup of cancers). Such genes are biologically informative genes. Unfortunately, we have failed to locate any study dealing with this important issue. None of the methods discussed earlier can be used easily to identify “multiple-class specific” marker genes. In principle, it may be possible to use methods in the filter category, but it will involve extensive computation. For example, due to the two-class discriminant nature of statistics, such as t-statistic (that is frequently used), further strategies, e.g., one-versus-one (OVO), one-versus-all (OVA), are required to apply these methods in identification of multiple-class specific marker genes. Consequently, to find useful biomarkers, a time-consuming procedure considering all possible combinations of classes has to be performed. Pavlidis and Nobel have proposed an ANOVA and template matching based approach [19] to identify such multiple-class specific biomarkers. But as explained above, to find genes with multiple-class specific signatures, we need to try all possible combinations of classes, which would demand considerable computation. Also, another interesting but time consuming procedure has been proposed in [20] to assign significance to statistically defined expression patterns.

On the other hand, for methods in the *embedded* and *wrapper* categories, although we can get better predictive performance, the interactions between the biomarkers and the diseases may not be easy to interpret/understand due to the non-linear nature of interactions. Moreover, to get multiple-class specific biomarkers, we shall still require some post-processing, which may not be easy. Finally, there may be some biomarkers for some specific groups of diseases, but such a method may not recognize/identify those because such methods are driven by minimization of classification error. Hence, there is a need for developing methods/algorithms that can find genes with multiple-class specific signatures.

Here we propose a novel gene selection index, *Group Marker Index* (GMI), which can identify *de novo* in a single process both single- and multiple-class specific signature genes (both are called group specific genes). It is computationally efficient in the sense that it does not require computation of the index for all possible subsets of classes. For a *K*-class data set, GMI is evaluated *K*-1 times rather than for *2^K^-2* combinations. GMI is a distribution-free method, which shares the advantage of non-parametric methods and is not influenced by the lack of knowledge about the distribution of data. Furthermore, we use GMI with a repeated random sampling procedure to select candidate marker genes in a more reliable manner. Here a permutation procedure is used to assess the significance of selected marker genes. We have used four multiple-class microarray data sets, which are Small Round Blue Cell Tumors (SRBCT) [21], Leukemia [22], Central Nervous System (CNS) [23], and Lung Cancer [24], to demonstrate the effectiveness of the proposed method. We demonstrate that genes identified by the proposed scheme participate in several important biological processes. Scatter-plots of the identified group specific genes for these data sets also exhibit good discriminating power among classes. Although we could not find any method in the literature addressing this problem, just for the sake of comparison, we have proposed and used an algorithm using a template-based gene selection scheme.

## Results

### Significance of genes selected by using GMI

In this study, we develop a gene evaluation index named “Group Marker Index (GMI)”, to select biologically significant genes for both two- and multiple-class cancer discrimination problems. Given expression profiles on *K* different types of cancers (diseases), our GMI-based algorithm tries to discover subsets of the cancers/diseases, if present, that can be discriminated from the remaining ones using gene expressions, as well as, the discriminatory genes. If a gene is highly expressed for a subset containing *n* classes (*n* = 1, 2, …, *K*-1) compared to the remaining (*K*-*n*) classes, then we call that gene a *level-n* discriminatory gene. This set of cancer classes with higher expression values is called the “upper group”. On the other hand, the set of remaining classes with lower expression values is called the “lower group”. The GMI algorithm finds, all *level-n* (*n* = 1, 2, …, *K*-1) genes, if exist. The detailed description of the algorithm is provided in the *Materials and Methods* section.

**Figure 1:**
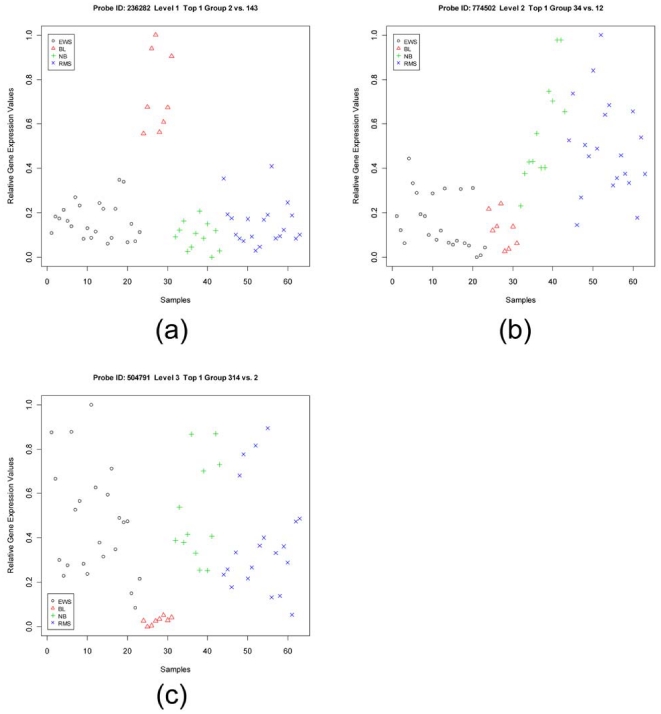
Scatter-plots of the top most gene of each level in the SRBCT data set. Panels (a), (b) and (c) are the scatter-plots of the top most gene of level-1, level-2, and level-3, respectively. The top most genes are WAS (236282), PTPN12 (774502) and GSTA4 (504791), respectively. There are four classes in the SRBCT data set: Ewing sarcomas (EWS), Burkitt lymphomas (BL), neuroblastomas (NB), and rhabdomyosarcomas (RMS).

The quality of genes selected by using GMI is demonstrated here in two significant ways. First, for each of the four data sets, we present visual assessments of the quality of the top most level-*n* gene for every level-*n* selected by GMI. We use a scatter-plot to show the distribution of gene expression values for the top most level-*n* gene. For the scatter-plot the y-axis expresses the observed gene expression values (normalized in [0,1]) and the x-axis indicates the number and identification of samples in a data set. The samples in different classes are represented by different symbols and colors, which help illustrate the discriminating power of (each) individual GMI-selected gene. For example, each sub-panel in Fig. 1 and Figs. S1, S2, S3 displays a top most level-*n* gene for different level *n* in the different data sets. As expected, each such gene appears with high gene expression values in the samples from *n* classes (i.e., the upper group, *infra* at Computation of GMI), but with low gene expression values in the samples of the remaining classes/subgroups (i.e., the lower group). Also, every top most level-*n* gene demonstrates good separation and low overlap between samples belonging to upper and lower groups.

In addition, we have further tabulated (Table 1 and Tables S1, S2, S3) the following basic information for the top 10 genes, which are individually selected using GMI from each level of discrimination: probe name, official gene symbol, class-labels in upper group and lower group, frequency of appearance of the gene in the list of top 10 genes, averaged GMI value, *p*-value and *q*-value obtained from the permutation test mentioned in the *Materials and Methods* section. These tables further provide us with several interesting conclusions: (a) We find that all GMI-selected genes have very low *p*- and *q*-values (in many cases it is so low that it is represented as zero) suggesting that these genes carry statistically significant multiple-class specific signatures. (b) For a given level of discrimination, the genes with quite high GMI values and appearing with high frequencies are considered better group-specific biomarkers. (c) We use the GMI value to represent a measure of discrimination for a gene between upper and lower groups. Thus, a gene with a higher GMI value implies its higher discriminating power between upper and lower groups. This information can be used for comparison of discriminating power of genes at different levels in the same data set. As demonstrated by the GMI values in Table 1 for the SRBCT data set, the discriminating power of level-2 genes is weaker than that of level-3 genes, and the discriminating power of level-3 genes is weaker than that of level-1 genes. Also as shown by the GMI values set forth in Table S3 for the Lung Cancer data set, the discriminating power of level-3 and level-4 genes is weaker than that of level-1 and level-2 genes. In addition, such phenomena can also be validated by the scatter-plot of the top most gene of each level for all data sets (Fig. 1 and Figs. S1, S2, S3). For example, *WAS*, the top most level-1 gene in the SRBCT data set, has a GMI value of 2.98 which is higher than the GMI value, 1.02, of *PTPN12*, the top most gene of level-2 in the same data set. From Fig. 1, we can observe that the discriminating ability of the *WAS* gene is much stronger than that of the *PTPN12* gene because the *WAS* gene has no overlapped sample between upper and lower groups, whereas the *PTPN12* gene has some overlapped samples. Hence, the visual illustration of the top most genes is consistent with the corresponding GMI values. (d) The GMI values can provide an objective assessment of the discriminating power even between data sets. For example, *VAMP2*, the top most level-1 gene in the Lung Cancer data set, has a GMI value of 7.19, showing discriminating power which appears even stronger than *WAS*, the top most level-1 gene in the SRBCT data set. Figure 1 and Figure S3 also reveal the stronger discriminating power of *VAMP2* over *WAS* because *VAMP2* also has no overlapped samples between upper and lower groups, and gets a bigger numerator for computing GMI (a higher value of the closest separation between a class in the upper group and a class in the lower group; i.e., the difference between the mean values of classes C*_n_*_(*s*)_ and C*_n_*_+1(*s*)_, or μ*_Sep_*_  = _ μ*_n_*_(*s*)_ – μ*_n_*_+1(*s*)_ in Eq. (1) mentioned in the *Materials and Methods* section). Our results demonstrate that GMI is very effective in identifying group-specific marker genes with clear discriminating power and we could obtain a robust list of candidate genes by conducting the repeated random splitting procedure.

**Table 1:** Summary of top 10 genes of each level selected by GMI in the SRBCT data set.

Level	Probe ID	Gene Symbol	Upper Group	Lower Group	Freq.	Ave. GMI value	p-value	q-value
1	236282	WAS	2	143	100	2.98	0	0
	770394	FCGRT	1	432	99	4.98	0	0
	241412	ELF1	2	143	91	2.45	0	0
	814260	FVT1	1	234	76	3.65	0	0
	377461	CAV1	1	432	57	2.37	0	0
	784224	FGFR4	4	213	53	2.43	0	0
	812105	MLLT11	3	412	52	2.57	0	0
	1435862	CD99	1	432	50	2.14	0	0
	183337	HLA-DMA	2	413	42	1.78	0	0
	796258	SGCA	4	231	37	2.02	0	0
2	774502	PTPN12	34	12	95	1.02	0	0
	365826	GAS1	14	32	75	0.80	0	0
	784593	RND3	34	12	72	0.75	0	0
	812965	MYC	21	43	64	0.72	0	0
	789182	PCNA	23	41	54	0.64	0	0
	859359	TP53I3	43	12	44	0.62	0	0
	714453	IL4R	42	13	38	0.57	2.17E-06	2.17E-06
	82903	(EST)	21	43	37	0.54	2.17E-06	2.17E-06
	159455	PLD3	41	32	34	0.53	2.17E-06	2.17E-06
	308163	YAP1	41	32	32	0.53	2.17E-06	2.17E-06
3	897164	CTNNA1	134	2	100	1.40	0	0
	504791	GSTA4	314	2	100	1.89	0	0
	897788	PTPRF	134	2	100	1.40	0	0
	295985	CDK6	342	1	86	1.52	0	0
	810057	CSDA	142	3	69	1.19	0	0
	21652	CTNNA1	143	2	61	0.85	0	0
	51320	DBNDD1	341	2	50	0.73	0	0
	212542	PBX1	143	2	48	0.59	0	0
	813742	XPO6	341	2	38	0.80	0	0
	741831	PLTP	143	2	28	0.54	2.17E-06	2.17E-06

Ewing sarcomas (EWS), Burkitt lymphomas (BL), neuroblastomas (NB), and rhabdomyosarcomas (RMS) are represented as Group 1 to Group 4 in order.

In addition, the conventional biomarkers for cancers are usually the level-1 genes with single tissue specific expression patterns. The protein products of those marker genes are usually receptors or proteins expressed in the cell surface. GMI can also identify the known biomarkers. For example, *FGFR4*, the level-1 gene highly expressed in rhabdomyosarcomas (RMS) class, is ranked within the top 10 level-1 genes in the SRBCT data set (Table 1). Multiple studies have reported that *FGFR4* is highly expressed in RMS [25]-[27] and the mRNA expression level of *FGFR4* is correlated with its protein level [25], [26], [28]. Furthermore, it has been shown that mutationally activated *FGFR4* acts as an oncogene [29]. Another level-1 gene ranked within the top 10 for the SRBCT data set, *CD99*, is highly expressed in Ewing sarcomas (EWS) class. It has been reported that CD99 is highly expressed in all EWS and the engagement of CD99 with anti-CD99 monoclonal antibodies would induce massive apoptosis as well as reduce malignant potential of EWS cells [30]. The deletion of *CD99* expression in human EWS cell lines would reduce their abilities of tumorigenesis and metastasis [31]. Moreover, the engagement of CD99 improves the efficiency of the conventional chemotherapeutic agents and reduces tumor growth along with a significant delay of metastasis [32]. These are some of the examples to demonstrate that GMI is capable of identifying the known biomarkers as well as the special type of group biomarkers.

### Comparison with template-based method

For a fair comparison of our GMI method with the existing methods for similar purpose, we have selected the most widely used template-matching based method and adapted it using the same iterative procedure as followed for GMI. We call this method as the *template-based method* (TBM). The detailed steps of the template-based method are described in the *Materials and Methods* section. First, we have used TBM to select group specific genes in the SRBCT and CNS data sets. Although, for the CNS data set, GMI can identify good level-2 discriminatory genes, it cannot identify good level-2 discriminatory genes for the SRBCT data set (please refer to Table 1 and Table S2). So for the SRBCT data set we want to check if there are useful level-2 discriminatory genes and GMI cannot identify those. For the CNS data set, we want to check whether GMI has already found good level-2 and level-3 discriminatory genes. In other words, we want to check if TBM can identify better genes. For each case, we have compared the top 10 genes identified by GMI and TBM.

Comparing the top 10 level-2 genes in the SRBCT data set (Table 2), we find that both GMI and TBM select *PTPN12* (774502) as the top most gene. Therefore, there may be no better level-2 gene in the SRBCT data set. To further check whether GMI miss any good level-2 gene, we focus on those genes which are identified by TBM but not by GMI. Between the two gene lists, there are four common genes. With a careful inspection of scatter-plots of the TBM-selected unique genes, we find that for each of such genes there is a substantial overlap between the upper and the lower groups. For a few of the GMI-selected unique genes, a similar situation arises. Thus to make a better objective evaluation of the two lists, for each gene in Table 2, we have performed a leave-one-out cross validation (LOOCV) with the nearest neighbor classifier (NNC), which enables us to assess the discriminating power of each gene for the two groups of diseases. The resultant accuracies are tabulated in the last column of Table 2. On the average, the GMI-selected unique genes are superior to the TBM-selected unique genes. One GMI-selected unique gene, Probe ID  =  365826 (ranked the second by GMI) achieves the highest accuracy of 0.9048! On the other hand, Probe ID  =  841620, which is ranked the second and selected only by TBM, achieves the least accuracy of 0.6508! This suggests that GMI selected genes are better discriminator; however, as we have mentioned earlier, finding genes suitable for designing classifiers is not the objective of this study.

**Table 2:** The comparison of top 10 level-2 genes selected by GMI and TBM in the SRBCT data set.

Probes	GMI Mean Order	GMI Rank	GMI Freq.	TBM Rank	TBM Template	TBM Freq.	LOOCV NNC Acc.
774502	(34)(12)	1	95	1	(34)(12)	83	0.8413
365826	(14)(32)	2	75	33	(14)(23)	7	0.9048
784593	(34)(12)	3	72	19	(34)(12)	16	0.7778
812965	(21)(43)	4	64	4	(12)(34)	65	0.7143
789182	(23)(41)	5	54	5	(23)(14)	50	0.8730
859359	(43)(12)	6	44	13	(34)(12)	25	0.8889
714453	(42)(13)	7	38	8	(24)(13)	40	0.7460
82903	(21)(43)	8	37	32	(12)(34)	7	0.7460
159455	(41)(32)	9	34	22	(14)(23)	12	0.8254
308163	(41)(32)	10	32	24	(14)(23)	11	0.7302
841620	(13)(42)	12	27	2	(13)(24)	77	0.6508
789204	(23)(41)	16	21	3	(23)(14)	71	0.7460
841641	(13)(42)	11	28	6	(13)(24)	47	0.7778
809557	(23)(14)	18	17	7	(23)(14)	40	0.7460
782811	(23)(14)	33	6	9	(23)(14)	36	0.6984
47542	(23)(41)	15	22	10	(23)(14)	35	0.7460

TBM: Template-based method.

Ewing sarcomas (EWS), Burkitt lymphomas (BL), neuroblastomas (NB), and rhabdomyosarcomas (RMS) are represented as Group 1 to Group 4 in order.

Table S4 reveals that for the CNS data set in the lists of top 10 level-2 genes identified by GMI and TBM there are 8 common genes; while for the level-3 genes there are 5 common genes (Table S5). Most of the genes common to both methods are on the top of the TBM gene list. These findings imply that there may not be better level-2 and level-3 discriminatory genes in the CNS data set. Also, the higher agreement between the gene lists identified by GMI and TBM in this data set with more balanced distribution of number of samples over classes may indicate that difference in sample sizes between classes can affect the gene selection.

To investigate the effect of variation of sample sizes between classes, we have compared the gene lists produced by GMI and TBM on Leukemia and Lung Cancer data sets. All classes in the Leukemia data set have comparable sample size. On the other hand, the Lung Cancer data set has one class with a relatively large sample size. We compare the top 10 genes for these two data sets. For the Leukemia data set, considering the level-2 genes we find that seven of the top 10 genes are common in the two lists. Moreover, both lists have the same genes at the top (Table S6). This high percentage of common genes in the two lists *might* be taken as an indicator that TBM would identify genes similar to those identified by GMI in cases where there is not much variation between sample sizes from different classes.

**Figure 2:**
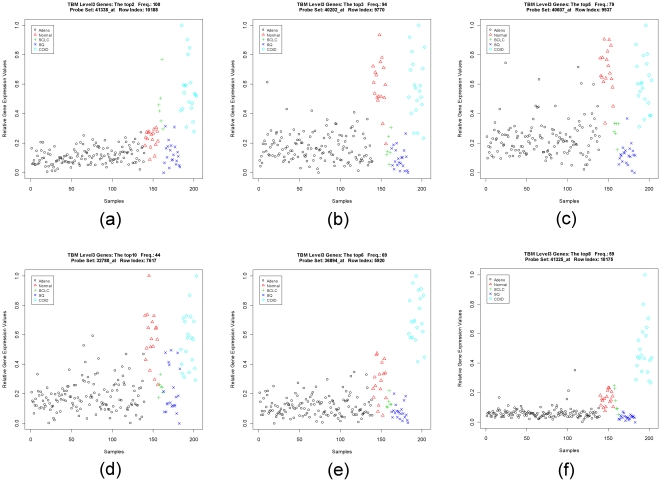
The level-2-like and level-1-like genes ranked within top 10 level-3 genes by template-based method in the Lung Cancer data set. Panels (a), (b), (c) and (d) are the scatter-plots of the level-2-like genes. Panels (e) and (f) are the scatter-plots of the level-1-like genes.

For the Lung Cancer data set, we consider both level-2 and level-3 discriminatory genes. In the case of level-2 genes, we have three common genes in the top 10 genes identified by GMI and TBM (Table S7). In order to assess the quality of the genes identified by GMI and TBM, we have compared the scatter-plots of those genes, which are identified by only one of the methods. A careful inspection of the scatter-plots suggests that the genes identified only by TBM are not better genes than those identified only by GMI for the purpose of discrimination (see File S1). Furthermore, *TAGLN3* (32650_at), which is ranked by GMI as the top most level-2 gene with the highest frequency of selection, is not in the top 10 gene list selected by TBM. But the scatter-plot of *TAGLN3* (Figure S3b) reveals that it is definitely a good level-2 gene. Concordant results are also revealed by the single gene LOOCV accuracy as depicted in Table S7, where *TAGLN3* achieves the perfect accuracy. While comparing the level-3 discriminatory genes, we have found that there are three common genes between the lists of top 10 genes identified by GMI and TBM (Table S8). Four of the top 10 genes (2^nd^, 3^rd^, 5^th^ and 10^th^) selected by TBM appear much like level-2 genes (see Figs. 2*a*-*d*). The top sixth and eighth genes selected by TBM appear more like level-1 genes (see Figs. 2*e*–*f*). Unlike genes selected by TBM, the scatter-plots of the top genes identified by GMI reveal that these genes are good level-3 discriminatory genes. On the average, the single gene LOOCV accuracies of GMI-selected unique genes are higher than the accuracies of TBM-selected unique genes.

**Figure 3:**
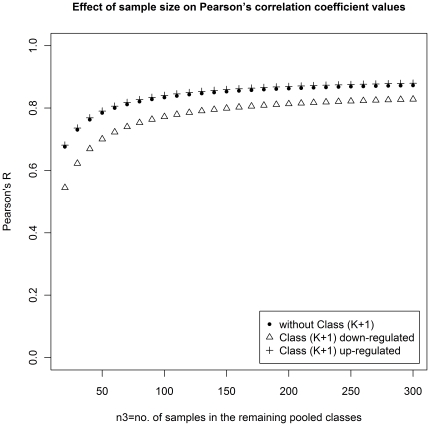
Effect of sample size on Pearson's correlation coefficient values.

We find that when the class sizes are balanced, more genes are found common in the top 10 level-*n* genes produced by GMI and TBM. When the class sizes are widely different, the lists of top 10 level-*n* genes are significantly different. And GMI is found to identify better discriminatory genes. A natural question arises, why? A possible reason for this may be the fact that correlation value for a gene with the designated template (used in TBM) is quite sensitive to the relative sizes of different classes in the training data. For example, even if the sizes of different classes remain the same, but the number of samples from one class keeps on increasing, the correlation value exhibit a monotonic behavior. This is illustrated in Fig. 3. Suppose in a *K*-class problem, class 1 has *n_1_* samples, class 2 has *n_2_* samples and the remaining classes together have *n_3_* samples. Suppose for a gene *g*, all samples from class 1 and 2 are highly expressed (with gene expression value of 1), while for the other classes the gene *g* is unexpressed (i.e., the gene expression value is zero). In this case, the correlation for the gene *g* with the ideal vector for class 1 (the ideal vector will have expression of 1 for class 1 and 0 for all other classes) will be 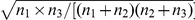. It can be easily seen that as *n_3_* increases with more and more unexpressed samples keeping the nature of the *n_1_* and *n_2_* samples from class 1 and 2 unaltered, the correlation goes to 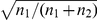. The filled circle in Fig. 3 illustrates this when *n_1_* = 60, *n_2_* = 15 and *n_3_* varies from 20 to 300. Note that, when *n_3_* = 20, the correlation value is 0.68 but that increases to 0.85 when *n_3_* becomes 140, although neither the expression profiles for class 1 and class 2 change nor the expression profile of the remaining classes changes, and for a multiple-class data 140 is not a big number. Thus, the template-based method, which selects genes based on correlation value, sometimes is not sensitive enough to reflect the desired behavior of samples. This will lead to improper ranking.

Following the same experimental design, we add one extra class (class (*K*+1)) with only five samples, which is either up-regulated or down-regulated. The template is so set that samples in class 1 and class (*K*+1) are highly expressed while unexpressed in the remaining classes. When class (*K*+1) is up-regulated, the tendency of the resultant correlation values (shown by the symbol “+” in Fig. 3) is almost the same as that of the *K*-class case. In case, class (*K*+1) is down-regulated, the nature of the resultant correlation values (shown by triangles in Fig. 3) is still similar to the previous *two* cases with correlation values in a slightly lower region. In these two (*K*+1)-class cases, most samples are unexpressed, which are contributed by the pool (class 3 to class *K*) with *n_3_* samples. Therefore, it has practically no effect when we set class (*K*+1) up-regulated. On the other hand, there is a little influence when class (*K*+1) is down-regulated. However, as the number of samples increases, the impact becomes less and less.

The level 2-like genes selected by TBM in the Lung Cancer data set (Figs. 2a–d) are good examples to show that this effect indeed happens in real data. All of those four genes are selected based on the template with high expression for classes 2, 3, and 5 (please refer to Table S8). However, the Lung Cancer data set is quite unbalanced in the number of samples between different classes. There are only six samples for class 3, which contributes a little in computing the correlation value of TBM (only four samples are randomly selected during the repeated random splitting procedure). Therefore, irrespective of whether class 3 is highly expressed or unexpressed, the contribution of class 3 in correlation may not be significant (Figs. 2*b*–*d*). Even for class 2 (Normal) which has 17 samples, the selection by TBM suffers from the same problem (Fig. 2a). Similarly, the level 1-like genes (Figs. 2*e*–*f*) can also support this effect.

### Biological Relevance of some level-2 and level-3 biomarkers

#### CNS data set

The first 4 genes in level-2 are highly expressed in human cerebellar tumors and malignant glioma (i.e., Ncer and MGlio; upper group representing 4 and 2) and are practically unexpressed in the rest of the classes (lower group consisting of 1, 3 and 5). Interestingly, six of the top genes in level-2 (*PRUNE2*, *TIMP4*, *TMOD1*, *ADORA1*, *NEUROD1* and *C1orf61*/*Croc4*) with GMI score ranging from 2.38 to 1.17 are primarily, if not solely, involved in cytoskeleton maintenance. Perturbation in the expression levels of these genes is likely to affect morphological, structural and functional integrity of the cell. For example, *PRUNE2* gene is over-expressed in prostate cancer and down-regulates Rho-A and Rho-C that are involved in actin polymerization and oncogenic transformation [33], [34]. TIMP4 inhibits tumor progression by inhibiting cell matrix degradation by endopeptidase MMP-2 [35]. TMOD1 regulates actin filament dynamics [36]. On the other hand, C1orf61/Croc4 positively controls c-fos activity and the later one up-regulates actin expression [37], [38]. Moreover, NEUROD1, a transcriptional factor, controls transcription of cytoskeletal genes [39]. In cancer, one of the frequently affected pathways is the cytoskeletal structural organization and the genes in this pathway are dys-regulated but not necessarily mutated. For example, cofilin expression is frequently increased in glioblastoma and ovarian cancer [40], [41] whereas cortactin is often over-expressed in breast cancer and squamous carcinoma of head and neck [42], [43]. Literature search revealed that none of the level-2 genes are ever mutated in any cancer but rather work to protect the cytoskeletal integrity of the cell. Over-expression of these genes in human cerebellar tumors and malignant glioma may reflect as an innate attempt of the cell to counteract the process of tumor transformation.

Earlier we proposed GDI system (Gene Dominant/Dormant Index) that selects two specific types of gene, i.e., dominant and dormant genes wherein the former is up-regulated in one specific class and down-regulated in the remaining classes, and the latter is down-regulated in one specific class but up-regulated in the rest of the classes [1]. Although different cancers, according to their tissue of origin, do differ in their mode of action through regulation and/or dys-regulation of various physiological pathways, they do also share some common genes/pathways in the formation of cancers. In that sense, GMI-discovered genes that are up-regulated in more than one-class of cancer (here we can also termed them as *co-dominant*) may provide additional biological insights of how various cancers might be related and probable molecular pathways involved in them.

In case of the level-3 genes, it appears that they are functionally diverse. However, the first two genes share a common cytoskeletal pathway. For example, FEZ1 (GMI score 3.95), highly expressed in the group {4, 2, 5}, is a brain specific cytoskeletal regulatory protein associated with microtubule and in various tumors it is either deleted and/or mutated [44]. But here in malignant glioma, human cerebellar tumor and primitive neuroectodermal tumor (PNET) the expression of FEZ1 is increased signifying a failed attempt of the cell to contain the tumor growth. Recent study shows that FRG1 (GMI value 2.07, up-regulated in group {1, 4, 3}) a multifunctional protein, specially binds to F-actin and its misregulation leads to facioscapulohumeral muscular dystrophy (FSHD) [45]. HMGN2 with GMI value of 1.11 (up-regulated in group {1, 3, 5}) is a highly conserved nucleosomal protein involved in unfolding higher-order chromatin structure and acts as an impediment for cell migration [46], [47]. VAT1 with GMI value of 1.14 (up-regulated in group {3, 1, 2}) controls the storage and release of neurotransmitters in the nerve terminal [48]. Importantly, none of these top-5 genes in both level-2 (except *PRUNE2*) and level-3 are ever reported as biomarkers (over-expressed and/or under-expressed) for any type of cancer. In that sense, our analysis demonstrates that these *co-dominant* genes may be used as unique signature genes in defining the respective tumor groups.

#### Lung Cancer data set

Level-2 and level-3 discriminatory genes in the Lung Cancer data set are quite interesting. As in the case for CNS data set, the four top level-2 discriminatory genes with GMI values ranging from 3.45 to 1.58 are primarily involved in regulation of cytoskeleton that controls vesicular trafficking in golgi bodies [49]. These genes are basically *co-dominant* in classes 5 and 3, representing pulmonary carcinoids (COID) and small cell lung cancer (SCLC), respectively. TAGLN3 associates with and regulates F-actin, α-tubulin, tau and MAP2 [50]. CRMP1 is a phosphoprotein and controls microtubules [51]. NCAM1 is a classical cell adhesion molecule that interacts with a number of cytoskeletal proteins and regulates cell architecture. INSM1 gene encodes a zinc finger DNA-binding domain and a putative prohormone domain. This gene is a sensitive marker for neuroendocrine differentiation of human lung tumors [52]. Interestingly, INSM1 acts as a transcriptional repressor of NEUROD that is involved in regulating cytoskeleton genes [53]. It is important to note that four of the first five genes in level-2 (TGLN3, CRMP1, NCAM1 and SCAMP5) are supposed to be neuron-specific [54]-[57]. But our analysis shows that these neuron-specific genes are aberrantly expressed in COID and SCLC class of lung tumor, most likely as a result of mis-functioning of SWI/SNF complex [58]. The fact that the top 4 level-2 discriminatory genes both in CNS and Lung Cancer data sets are involved in the same molecular pathway might be a pure coincidence or else it could be that pathways of cancer, irrespective of types, are frequently involved dys-regulating genes associated with cytoskeleton architecture.

On the other hand, the top 6 level-3 discriminatory genes are basically involved in calcium signaling; however, they bear quite a low GMI values ranging from 1.25 to 0.34. These genes, except SEC14L1, are primarily *co-dominant* in the classes 4, 1 and 2, i.e., squamous cell carcinomas (SQ), lung adenocarcinomas (Adeno) and normal lung (Normal). S100A11 and S100A10 are calcium binding proteins and their increased expression are often observed in colorectal and prostate cancer, including non-small cell lung cancer [59], [60]. VAMP8 is involved in calcium-dependent exocytosis process and SEC14L1 is a membrane trafficking protein, and none of them is linked to cancer pathways. On the other hand, LGALS3, an IgE-lectin binding protein, is often abundantly expressed in thyroid cancer, hepatocellular carcinoma and in non-small cell lung cancer [61], [62]. FAM38 is involved in intracellular calcium release [63]. Calcium signaling plays very important role in cancer in mediating angiogenesis steps such as invasion, adhesion and tumor cell migration. In many cancers, calcium signaling genes are up-regulated. The fact that the aforementioned genes are also up-regulated in normal lung (class 2) indicates that intact calcium signaling pathway may be necessary for tumorigenesis process in these cancer subgroups.

In a nutshell GMI method establishes that cytoskeleton regulating genes are aberrantly expressed in pulmonary carcinoids (COID) and small cell lung cancer (SCLC) whereas calcium signaling pathway genes are active in the rest of the classes, i.e., squamous cell carcinomas (SQ), adenocarcinomas (Adeno), and normal lung (Normal).

Typical microarray data analysis finds markers that can differentiate one class of cancer from the others, while GMI can find markers, which exhibit similar expression pattern in a group of cancers and can distinguish one group of cancers from another group of cancers. Thus, the importance of our GMI algorithm is in identifying *de novo* group specific genes in an automatic manner. When we have samples from multiple cancers/diseases with less knowledge about the relationship between them, GMI could be very useful to discover a group (subset) of cancers, if exists, that can be discriminated from the remaining set of cancers using expression pattern of one or more genes. This in turn can help us to understand the relationship between subsets of cancers/diseases groups via functional analysis of the GMI-identified group specific genes. Further, existence and discovery of such group markers open up the possibility of finding common drug targets for different diseases as well as possibility of using drugs designed for one disease to cure another disease.

### KEGG pathway analysis

After analyzing the relationship between a single gene and multiple diseases, we attempt to explore the relationship between the groups of discriminatory genes and diseases. For this, instead of considering level-1 discriminatory genes, which may be identified by all gene selection methods, we focus on the level-2 discriminatory genes first. We try to identify related biological pathways in which the level-2 discriminatory genes are involved by cross-referencing to the Kyoto Encyclopedia of Genes and Genomes (KEGG) database [64]-[66].

We take the advantage of the abundant number of genes in the Leukemia and Lung Cancer data sets in the data selection for pathway analysis. For the Leukemia data set, we consider all level-2 genes with *p*-values smaller or equal to 0.0001. Subsequently, we divide these level-2 genes into three different gene lists based on the composition of the upper group. The numbers of genes, actually probe sets, in each list were: 712 for the upper group composed of acute lymphoblastic leukemia (ALL) and mixed-lineage Leukemia (MLL), which we call ALL-MLL list; 3 for the upper group composed of ALL and acute myelogenous leukemia (AML), which is termed as ALL-AML list; 100 for the upper group composed of MLL and AML (MLL-AML list). Next, we use the functional annotation tool provided by the database for annotation, visualization and integrated discovery (DAVID ver. 6.7) [67], [68] to cross-reference to the KEGG database (accepted default parameters).

For the ALL-MLL list, 709 probe sets that belong to *Homo sapiens*, are selected and the default background is used for the analysis. A total of 665 DAVID IDs are converted and 228 probe sets are involved in the KEGG pathway category. The output results provided by DAVID are summarized in Table 3. The identified pathways/annotation terms are tabulated in the first column (Term). The total number of genes (Count) and the corresponding percentage (%) in our gene list involved in each pathway along with the modified Fisher Exact *p*-value (p-value), and other enrichment quantitative measurements (Fold Enrichment, Bonferroni, Benjamini, FDR) are also included in Table 3. As shown in Table 3, there are four pathways with EASE Score, the modified Fisher Exact *p*-value, smaller than 0.01. These are spliceosome, B-cell receptor signaling, basal transcription factors and inositol phosphate metabolism pathways. Literature search revealed that these pathways are often impaired in ALL and MLL development [69]–[73]. On the other hand, if we consider EASE Score smaller than 0.05, then 15 pathways are identified. The full table with probe set lists is provided in Table S9. Most of these additional pathways are involved in cancer as evident by their name such as colorectal cancer pathways, base excision repair, mismatch repair, nucleotide excision repair, and more specifically involvement of chronic myeloid leukemia pathway is noteworthy. Interestingly, pathways like fatty acid metabolism, phosphatidylionositol signaling and ubiquitin mediated proteolysis that at first sight seemed to be remotely associated with ALL or MLL but are indeed found to be very much molecularly involved in the pathogenesis of ALL and MLL as revealed by literature search [74]–[77].

**Table 3:** Summarization of the identified pathways related to the level-2 discriminatory genes in the Leukemia data set.

Term	Count	%	p-value	Fold Enrichment	Bonferroni	Benjamini	FDR
ALL-MLL List							
hsa03040:Spliceosome	21	3.1579	5.38E-07	3.7171	8.23E-05	8.23E-05	6.44E-04
hsa04662:B cell receptor signaling pathway	14	2.1053	2.13E-05	4.1632	0.0033	0.0016	0.0256
hsa03022:Basal transcription factors	7	1.0526	0.0040	4.4605	0.4623	0.1868	4.7433
hsa00562:Inositol phosphate metabolism	8	1.2030	0.0095	3.3041	0.7685	0.3064	10.8272
hsa05210:Colorectal cancer	10	1.5038	0.0120	2.6551	0.8413	0.3080	13.4271
hsa03420:Nucleotide excision repair	7	1.0526	0.0126	3.5481	0.8565	0.2765	14.1065
hsa04910:Insulin signaling pathway	13	1.9549	0.0168	2.1477	0.9248	0.3091	18.3459
hsa03430:Mismatch repair	5	0.7519	0.0175	4.8484	0.9333	0.2871	19.1067
hsa05220:Chronic myeloid leukemia	9	1.3534	0.0178	2.6763	0.9363	0.2636	19.3979
hsa03410:Base excision repair	6	0.9023	0.0184	3.8233	0.9419	0.2477	19.9781
hsa05340:Primary immunodeficiency	6	0.9023	0.0184	3.8233	0.9419	0.2477	19.9781
hsa04660:T cell receptor signaling pathway	11	1.6541	0.0216	2.2716	0.9646	0.2620	23.0243
hsa00071:Fatty acid metabolism	6	0.9023	0.0312	3.3454	0.9922	0.3327	31.6244
hsa04120:Ubiquitin mediated proteolysis	12	1.8045	0.0414	1.9535	0.9985	0.3923	39.7743
hsa04070:Phosphatidylinositol signaling system	8	1.2030	0.0459	2.4111	0.9992	0.4014	43.0280
MLL-AML List							
hsa05221:Acute myeloid leukemia	4	4.4944	0.0055	10.6270	0.3252	0.3252	5.5876
hsa04662:B cell receptor signaling pathway	4	4.4944	0.0112	8.2182	0.5520	0.3307	11.0767
hsa04664:Fc epsilon RI signaling pathway	4	4.4944	0.0125	7.9021	0.5909	0.2577	12.2495
hsa04062:Chemokine signaling pathway	5	5.6180	0.0285	4.1201	0.8715	0.4013	25.9129

The contents of Tables 3-5 are the output results provided by DAVID. The first column (Term) contains the identified KEGG pathways. The second column (Count) indicates the total number of genes in our gene list which is involved in each pathway. The third column (%) shows the same information as shown by the second column but using percentage. The following columns (*p*-value, Fold Enrichment, Bonferroni, Benjamini, FDR) represent the modified Fisher Exact p-value and other enrichment quantitative measurements, respectively. For more detailed information, please refer to DAVID[67], [68].

Using the same procedure, 98 probe sets belonging to *Homo sapiens* and one probe set belonging to *Mus musculus* are selected for the MLL-AML list. These probe sets are mapped to 89 DAVID IDs and only 33 probe sets are involved in the KEGG pathway category. Befitting our method of analysis, the only pathway identified by the functional annotation tool with EASE Score smaller than 0.01 is the Acute Myeloid Leukemia pathway (KEGG entry ID: hsa05221). This attests that our method of analysis may be relevant in identifying the gene(s) signatures for distinguishing subtypes of cancer. Additional three pathways, B cell signaling, Chemokine signaling and Fc epsilom R1 signaling, which have passed the EASE Score criterion (*p*-value < 0.05) are also shown in the Table 3. These are all well documented pathways that are impaired in AML and/or MLL cancer [78]-[80]. Moreover, MLL is a special ALL group, which carries *MLL* gene translocation. The authors of the Leukemia data set [22] collected ALL and MLL samples from the individuals diagnosed as CD19^+^ B-precursor ALL without and with *MLL* translocation, respectively. Those well-known B cell marker genes, e.g., *CD19*, *CD81*, *CD79A*, *CD79B*, are all contained in the ALL-MLL list. Some other genes, which have been discussed by the authors [22] e.g., *IL7R*, *DNTT*, *TCF3*, *POU2AF1* and *SMARCA4*, are also listed in the ALL-MLL list. The rest of genes in the ALL-MLL list are related to B cell proliferation, phosphorylation, DNA replication, tumor development. On the other hand, the myeloid-specific genes e.g., *CCNA1*, *SERPINB1*, *RNASE3*, and some other genes that are discussed by the authors who published the data set e.g., *CD44*, *HOXA9*, *HOXA5*, *SPN*, *LGALS1*, *ANXA1*, *ANXA2*, are contained in the MLL-AML list. The only three genes exhibiting common up-regulated pattern in ALL and AML are *RYK*, *SCHIP1*, and *YESP1*. The long list of level-2 discriminatory genes for the ALL and MLL group suggests that between the three classes, the ALL and MLL group share more similar physiological properties. MLL represents mixed-lineage features yet we can find some similarities between MLL and AML. However, ALL and AML are distinguished from each other. In a nutshell, many of the level-2 discriminatory genes identified in our analysis for leukemia class-specific signatures are directly involved in pathways that lead to the development of leukemia. We believe that GMI would be helpful in understanding the relationship between unknown classes.

Furthermore, we find that the B cell receptor signaling pathway was identified by both ALL-MLL list and MLL-AML list from Table 3. To further investigate this pathway, we downloaded the figure of this pathway from KEGG [64]–[66] and labeled the level-2 genes using the same principle as used by DAVID [67], [68]. The modified figure is shown in Fig. S4. In Figure S4, those genes, which are relatively up-regulated in the ALL-MLL group, are labeled with red stars. On the other hand, those genes, which are up-regulated in MLL-AML group, are labeled with blue stars. We may interpret those red star genes as lymphoblastic genes because of the relatively up-regulated expression in both ALL and MLL but down-regulated expression in AML. Subsequently, those blue star genes can be treated as myelogenous genes because of their relatively lower expression in ALL. As summarized by KEGG [64]–[66], the activation of this pathway will involve in B cell proliferation, differentiation and Ig production as well as other processes. Thus, we find that many genes involved in this pathway are labeled with red stars (highly expressed in the CD19^+^ B-precursor ALL and MLL). We may interpret that the activation of *CD79A* (Igα) and *CD79B* (Igβ) with the co-simulators *CD81* and *CD19* triggers the activation of B cell signaling pathway in ALL and MLL. Furthermore, the co-inhibitor *LIRB3* (PIR-B) is also relatively down-regulated in ALL which could be treated as a positive factor for activating the B cell signaling pathway. Notably, the relatively lower expression of *Rac* in ALL may play an important role of lymphoblastic leukemia. In murine study, it has been demonstrated that *Rac* genes are important for appropriate positioning of hematopoietic stem cells (HSCs) within the bone marrow microenvironment. The deletion of both *Rac1* and *Rac2* murine alleles would lead to a massive egress of HSCs into the blood from the marrow [81], [82]. Thus, we may understand the role of B cell signaling pathway between those different groups of leukemia. This is an example to demonstrate how multiple level-2 (in general level-k) genes can be useful to interpret the observations and to understand the mechanisms behind. We believe that GMI algorithm will be a useful algorithm to the community.

In the Lung Cancer data set, we select all probe sets with *p*-value smaller than or equal to 0.0001 and group these probe sets into different gene lists for different compositions of the upper group. In the results shown in Table 4, there are five combinations in which we can identify related pathways (EASE Score < 0.05). These five combinations are Adeno-Normal, Adeno-SQ, Normal-SCLC, SCLC-SQ and Normal-COID. Each of these combinations belongs to the upper group. These pathways associated with SCLC-COID list are independently tabulated in Table 5. The full tables with probe set lists are also provided in Table S10 and Table S11. Out of these five combinations two combinations, Adeno-SQ and SCLC-SQ are important in order to molecularly distinguish four classes of lung cancer. In SCLC-COID group out of 27 pathways identified, 10 pathways are directly involved in cancer as their name suggests and additional pathways like splicesome, phosphatidylinositol signaling, calcium signaling and cell cycle pathways are also participatory in cancer process as discussed earlier. Importantly, the pathways like wnt, ErbB, MAPK, autophagy and Jak-Stat signaling are all well established in cancer development process. However, it is presently unclear as why Alzheimer's, Type II Diabetes, Long-term potentiation and Neurotrophin signaling pathways genes are up-regulated in SCLC-COID groups since their roles in cancer are not well founded. In the Adeno-SQ list, genes belonging to pathways of Ribosome, ECM receptor interaction and Focal adhesion were found to be up-regulated. Literature search revealed that all of these pathways are more or less compromised in Adeno-SQ [83]-[86].

**Table 4 pone-0024259-t004:** Summarization of the identified pathways related to the level-2 discriminatory genes in the Lung Cancer data set (Part I).

Term	Count	%	p-value	Fold Enrichment	Bonferroni	Benjamini	FDR
Adeno-Normal List							
hsa05416:Viral myocarditis	10	9.0909	1.17E-07	11.5516	1.17E-05	1.17E-05	1.30E-04
hsa05330:Allograft rejection	8	7.2727	1.60E-07	18.2258	1.60E-05	7.99E-06	1.77E-04
hsa05332:Graft-versus-host disease	8	7.2727	2.86E-07	16.8238	2.86E-05	9.55E-06	3.18E-04
hsa04940:Type I diabetes mellitus	8	7.2727	4.88E-07	15.6221	4.88E-05	1.22E-05	5.42E-04
hsa05320:Autoimmune thyroid disease	8	7.2727	1.93E-06	12.8653	1.93E-04	3.85E-05	0.0021
hsa04612:Antigen processing and presentation	9	8.1818	5.23E-06	8.8933	5.23E-04	8.71E-05	0.0058
hsa05310:Asthma	6	5.4545	2.00E-05	16.9689	0.0020	2.86E-04	0.0222
hsa04514:Cell adhesion molecules (CAMs)	9	8.1818	1.54E-04	5.5920	0.0153	0.0019	0.1711
hsa05322:Systemic lupus erythematosus	8	7.2727	1.58E-04	6.6276	0.0157	0.0018	0.1754
hsa04672:Intestinal immune network for IgA production	6	5.4545	2.68E-04	10.0428	0.0264	0.0027	0.2966
hsa00590:Arachidonic acid metabolism	6	5.4545	5.03E-04	8.7874	0.0490	0.0046	0.5564
hsa04640:Hematopoietic cell lineage	7	6.3636	5.18E-04	6.6757	0.0505	0.0043	0.5731
hsa04142:Lysosome	7	6.3636	0.0026	4.9069	0.2279	0.0197	2.8294
hsa04210:Apoptosis	5	4.5455	0.0199	4.7136	0.8660	0.1338	19.9982
hsa04610:Complement and coagulation cascades	4	3.6364	0.0492	4.7546	0.9936	0.2858	42.8973
Adeno-SQ List							
hsa03010:Ribosome	7	14.2857	4.68E-06	14.6121	0.0002	0.0002	0.0042
hsa04512:ECM-receptor interaction	6	12.2449	6.62E-05	12.9719	0.0025	0.0013	0.0598
hsa04510:Focal adhesion	4	8.1633	0.0888	3.6141	0.9708	0.6922	56.8582
Normal-SCLC List							
hsa04510:Focal adhesion	3	10.7143	0.0286	9.4869	0.4567	0.4567	20.0947
Normal-COID List							
hsa04142:Lysosome	5	3.9683	3.09E-02	4.1001	9.26E-01	9.26E-01	2.85E+01
SCLC-SQ List							
hsa03040:Spliceosome	12	12.5000	1.83E-08	9.6857	9.15E-07	9.15E-07	1.76E-05
hsa03030:DNA replication	8	8.3333	3.34E-08	22.6000	1.67E-06	8.36E-07	3.22E-05
hsa04110:Cell cycle	10	10.4167	2.20E-06	8.1360	1.10E-04	3.67E-05	0.0021
hsa03410:Base excision repair	6	6.2500	1.76E-05	17.4343	8.81E-04	2.20E-04	0.0170
hsa00670:One carbon pool by folate	3	3.1250	0.0100	19.0687	0.3955	0.0958	9.2400

**Table 5:** Summarization of the identified pathways related to the level-2 discriminatory genes in the Lung Cancer data set (Part II).

Term	Count	%	p-value	Fold Enrichment	Bonferroni	Benjamini	FDR
SCLC-COID List							
hsa04020:Calcium signaling pathway	55	2.0992	6.44E-06	1.8058	1.23E-03	1.23E-03	0.0080
hsa05223:Non-small cell lung cancer	24	0.9160	1.06E-05	2.5682	0.0020	1.01E-03	0.0132
hsa03040:Spliceosome	40	1.5267	1.01E-04	1.8344	0.0191	0.0064	0.1256
hsa04070:Phosphatidylinositol signaling system	27	1.0305	1.54E-04	2.1083	0.0289	0.0073	0.1909
hsa04722:Neurotrophin signaling pathway	39	1.4885	1.56E-04	1.8174	0.0294	0.0059	0.1941
hsa04210:Apoptosis	30	1.1450	1.89E-04	1.9926	0.0355	0.0060	0.2350
hsa05214:Glioma	24	0.9160	1.90E-04	2.2013	0.0357	0.0052	0.2363
hsa04010:MAPK signaling pathway	70	2.6718	1.95E-04	1.5149	0.0366	0.0047	0.2428
hsa05218:Melanoma	26	0.9924	1.97E-04	2.1160	0.0369	0.0042	0.2448
hsa05220:Chronic myeloid leukemia	27	1.0305	1.97E-04	2.0802	0.0370	0.0038	0.2452
hsa04110:Cell cycle	38	1.4504	4.09E-04	1.7566	0.0752	0.0071	0.5081
hsa04012:ErbB signaling pathway	29	1.1069	4.68E-04	1.9261	0.0855	0.0074	0.5806
hsa04930:Type II diabetes mellitus	19	0.7252	4.79E-04	2.3360	0.0874	0.0070	0.5937
hsa05210:Colorectal cancer	28	1.0687	6.01E-04	1.9261	0.1085	0.0082	0.7453
hsa05213:Endometrial cancer	20	0.7634	6.70E-04	2.2225	0.1201	0.0085	0.8297
hsa05200:Pathways in cancer	80	3.0534	7.56E-04	1.4094	0.1345	0.0090	0.9364
hsa05010:Alzheimer's disease	45	1.7176	0.0011	1.5953	0.1875	0.0121	1.3434
hsa05215:Prostate cancer	28	1.0687	0.0016	1.8179	0.2646	0.0169	1.9818
hsa04720:Long-term potentiation	23	0.8779	0.0017	1.9545	0.2790	0.0171	2.1081
hsa04914:Progesterone-mediated oocyte maturation	27	1.0305	0.0021	1.8142	0.3252	0.0195	2.5289
hsa04310:Wnt signaling pathway	41	1.5649	0.0026	1.5690	0.3949	0.0236	3.2188
hsa04114:Oocyte meiosis	32	1.2214	0.0028	1.6810	0.4135	0.0240	3.4154
hsa05222:Small cell lung cancer	26	0.9924	0.0031	1.7886	0.4524	0.0258	3.8464
hsa04622:RIG-I-like receptor signaling pathway	23	0.8779	0.0032	1.8719	0.4533	0.0248	3.8563
hsa05212:Pancreatic cancer	23	0.8779	0.0038	1.8459	0.5188	0.0288	4.6529
hsa04140:Regulation of autophagy	14	0.5344	0.0039	2.3114	0.5298	0.0286	4.7956
hsa04630:Jak-STAT signaling pathway	41	1.5649	0.0043	1.5285	0.5645	0.0303	5.2701

To summarize, KEGG pathway analysis establishes that there is as such no specific pathway(s) that can exclusively determine the subgroups of cancer. A plethora of pathways, shared and/or non-shared, is activated in various cancer subgroups. The level-*n* discriminatory genes found by GMI are important genes that play a major role in multiple cancer pathways.

## Discussion

In this study, we emphasize the important role played by multiple-class specific marker genes. We found that most available gene selection algorithms focus on or tend to identify single-class specific signature genes as marker genes. But, multiple-class specific markers may play important roles in biology besides construction of computational prediction systems. The lack of intuitive, easy-to-use methodologies for biologists inspired us to propose a novel gene selection algorithm based on an index called, Group Marker Index (GMI), which is efficient in the identification of both single- and multiple-class specific signature genes from microarray data. For the sake of comparison, a template-based method (TBM) is also formulated and used along with GMI. GMI differs from template-based methods in two aspects. First, to evaluate each gene, GMI uses the ordering of mean gene expression values for all classes that enables it to identify *de novo* certain combination of classes for each level of discrimination. It does not need to check all combinations of classes and hence it reduces drastically the computation overhead. We have demonstrated that GMI can identify robust and statistically significant marker genes for each level of discrimination using a repeated random-splitting procedure (Table 1 and Tables S1, S2, S3). Second, no prior knowledge is required for template assignment. It is possible for GMI to infer novel relationships between the studied classes/diseases. We have discussed the relevance/biological roles played by several level 2/3 genes. In nutshell, we observed selective dys-regulation of cytoskeleton regulating gene-network primarily at level-2 in both CNS and lung cancer. Secondly, we found that neuron-specific cytoskeleton genes are aberrantly expressed in COID and SCLC tumor of lung. Third, calcium signaling pathway genes are upregulated at level-3 of lung tumor. This method hitherto uncovered the importance of cytoskeleton genes and their use as class-specific markers for cancer diagnosis. In addition, we have also mapped a group of level 2/3 genes to the KEGG pathways. Both results exhibited concordant findings and implied potentially common properties between different classes or cancers. It suggests the possibility of identifying common drug targets between different diseases. It also opens up the possibility of using a specific remedy designed for one disease to cure another disease.

Second, GMI uses a non-parametric indicator, between-class-transition (BCT), to evaluate the discrimination between classes rather than evaluating the similarity between a template and gene expression values. An advantage of exploiting discrimination ability rather than similarity is that it ensures that the selected genes will be able to discriminate between subsets of classes at least to a reasonable extent. Inspired by the work in [19], [20] where authors used Analysis of Variance (ANOVA) along with the template-matching step for their purposes, here we have performed the ANOVA test between gene expression values and class labels for each gene. We find that a gene with a low ANOVA *p*-value often does not exhibit good discrimination ability between classes. Our experience shows that most genes in a multiple-class microarray data set are evaluated with small *p*-values by ANOVA test **(**See Fig. S5 **)**. Thus, for such a problem the main criterion used for gene selection is essentially the correlation value. Moreover, since TBM relies on correlation, it suffers from an additional problem (as already explained) while applying on the microarray data with unbalanced sample sizes over different classes. We have designed an example with unbalanced samples (Fig. 3) to demonstrate that the class with large sample size biases the resultant correlation value and the class with small sample size may not contribute much to the correlation. This is an inherent limitation of template based methods.

## Materials and Methods

### Data sets

#### SRBCT data set

It is a cDNA microarray data set with 63 samples from 4 classes of childhood small round blue cell tumors (SRBCT): 23 Ewing sarcomas (EWS), 8 Burkitt lymphomas (BL), 12 neuroblastomas (NB), and 20 rhabdomyosarcomas (RMS). Each sample is represented by 2308 genes. This data set is available at http://research.nhgri.nih.gov/microarray/Supplement/.

#### Leukemia data set

This Affymetrix high-density oligonucleotide array data set has 57 samples from 3 classes of leukemia: 20 acute lymphoblastic leukemia (ALL), 17 mixed-lineage leukemia (MLL), 20 acute myelogenous leukemia (AML), each with 12582 genes. This data set is available at http://www.broad.mit.edu/cgi-bin/cancer/datasets.cgi.

#### CNS data set

CNS is also an Affymetrix high-density oligonucleotide microarray data set containing 42 samples distributed over 5 different types of tumors of the central nervous system (CNS): 10 medulloblastomas (MD), 10 malignant gliomas (MGlio), 10 atypical teratoid/rhabdoid tumors (Rhab), 8 primitive neuro-ectodermal tumors (PNET), and 4 human cerebella tumors (Ncer). For this data set each sample is represented by 7129 genes. This data set is available at http://www.broad.mit.edu/cgi-bin/cancer/datasets.cgi.

#### Lung Cancer data set

In this Affymetrix high-density oligonucleotide array, we have 203 samples in 12600 dimensions. There are 5 classes: 139 lung adenocarcinomas (Adeno), 21 squamous cell lung carcinomas (SQ), 20 pulmonary carcinoids (COID), 6 small-cell lung cancer (SCLC), and 17 normal lung specimens (Normal). This data set can be obtained from http://www.pnas.org/content/suppl/2001/11/13/191502998.DC1/DatasetA_12600gene.xls.

### Preprocessing

For the Leukemia and CNS data sets, in the preprocessing step the gene expression values less than 100 are raised to 100 and gene expression values greater than 16000 are set to 16000. All gene expression values are then subjected to a base 10 logarithmic transformation. After that, the distribution of gene expression values in each sample is adjusted to zero mean and unit variance. For the SRBCT data set, we do not make any change to the gene expression values as that had already been preprocessed in the original data source [21]. For the Lung Cancer data set, we use the same preprocessed data as used in the original paper [24] without doing any additional preprocessing. For these four data sets, we adopt the same data preprocessing protocols as used in a previous study [1], [15]. In this study, the analysis is conducted using the R environment [87].

### Group Marker Index (GMI)

Our main objective is to develop a gene evaluation index, which we call “Group Marker Index (GMI)”, to select biologically significant genes for both two- and multiple-class discrimination problems. GMI builds on the Gene Dominant/Dormant Index (GDI) which was proposed in our earlier study. GDI is a gene evaluation index to select two specific types of genes, i.e., dominant and dormant genes defined in our previous study [1]. In a multiple-class microarray data set, a dominant gene has high gene expression values only in the samples from one specific class and low gene expression values in the samples of the remaining classes. Contrary to the dominant gene, a dormant gene has low gene expression values in only one specific class and high gene expression values in the rest of the classes. GMI is a further refinement of GDI in two aspects: First, irrespective of the number of classes, GDI will only evaluate two levels of discrimination (i.e., dominant and dormant conditions). GMI, on the other hand, will evaluate *K*-1 levels of discrimination where there are *K* classes in a pooled microarray experiment. Second, GDI uses the same equation of signal-to-noise ratio (SNR) to evaluate genes, which makes GDI sensitive to outliers, if any. We have avoided these deficiencies by utilizing a simple yet novel intuitive concept, referred to as Between-Class-Transition (BCT), as a further refinement to help identify overlapping classes.

### Between-Class-Transition (BCT)

Let 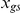
be the gene expression value for the *g* ^th^ gene in the *s* ^th^ sample, *g* = 1, 2, …, *G*; *s* = 1, 2, …, *S*. The *s* ^th^ sample is associated with a class label 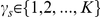. For each gene *g*, *g* = 1, 2, …, *G*, we sort the gene expression values of the *S* samples in descending order. In the sorted sequence, each gene expression value is associated with the class label of the corresponding sample. In this sorted sequence, if the class labels of two successive samples (gene expression values) are different, we count it as a between-class-transition (BCT). In this way, for each gene, we find the total number of BCTs.

### Illustration of BCT

We now illustrate BCT using a simple synthetic data set. Consider the gene expression values of a gene for 10 samples divided into two classes (first five samples belong to class 1 and the rest belong to class 2) as depicted in the left panel of Fig. S6. The sorted gene values are depicted in the right panel of Fig. S6. Here we have projected the samples on a vertical line and represented the two classes using two different symbols. In the right panel, the fourth sample from class 1 is sandwiched between two samples from class 2 and this adds two to the BCT count. In this way, the total number of BCTs is 5. An ideal marker gene in a two-class problem will have no outlier sample in either class, and the discrimination between the two classes will be very easy. In such a case the BCT value is only one (for the transition from one class to the other). In all other cases, where there are some outlier samples with gene expression values that overlap with the gene expression values of the samples from the other class, the BCT value will be larger than one. Note that, there could be BCTs even when there is no outlier in the true sense of the word, but the classes have overlap. With an increase in the number of overlapped samples, the BCT value will increase. Therefore, we can use the number of BCTs to help identify overlapped samples, and thus improve marker gene identification.

Consider a two-class problem, as an example. Suppose for a gene *g*, the samples from each class form compact clusters and the clusters are well separated except for the gene expression value of just one sample in one of the two classes, which is mixed with samples from the other class. In this case, the effect of the outlier on the standard deviation will depend on its location; in other words, how far the outlier is from the mean of the gene expression values among samples in that class. Thus, the effect of just one outlier (mixed sample) on the standard deviation could be moderate to severe. However, this is not the case with BCTs because the number of BCTs will not depend on the *location* of the outliers in the other class.

### Computation of GMI

**Figure 4:**
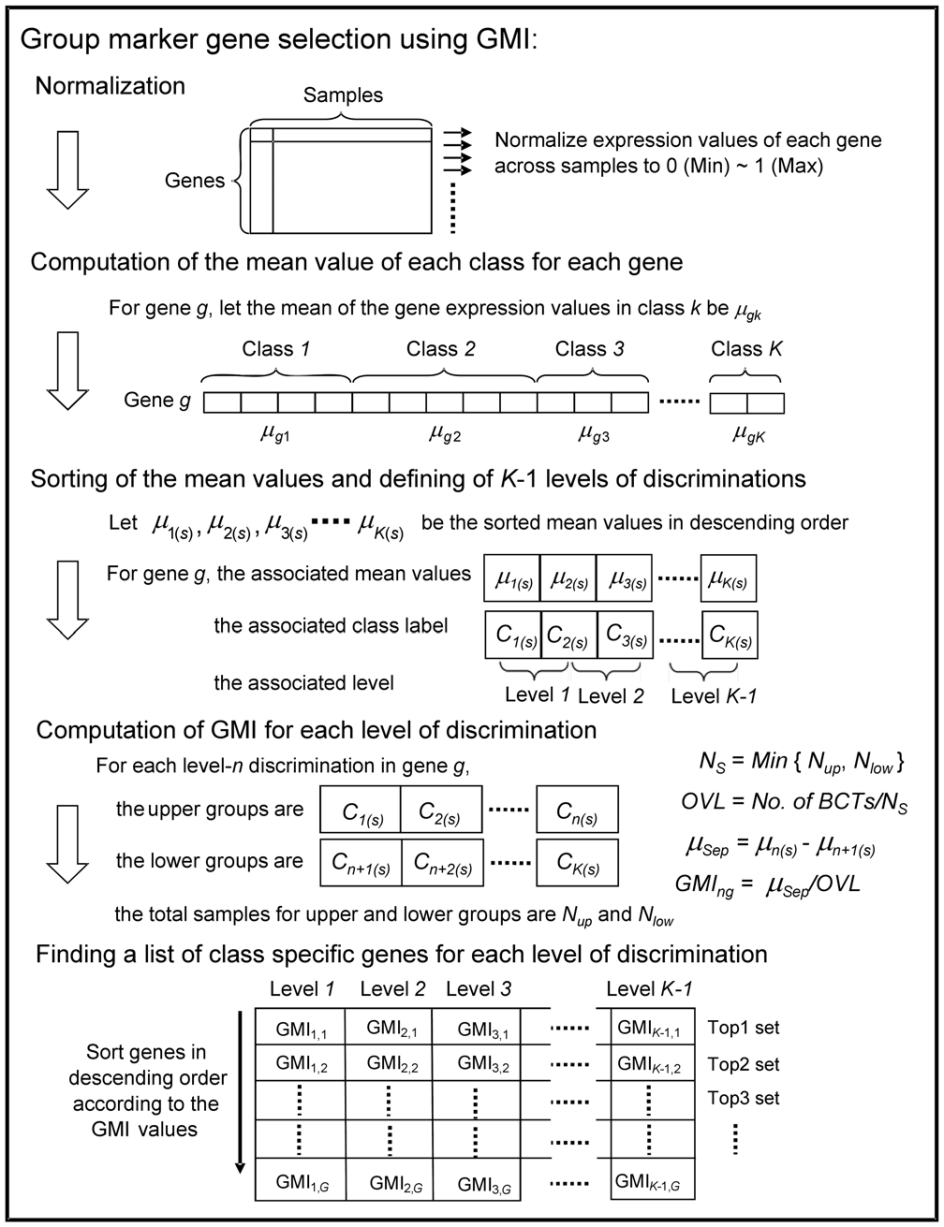
Steps involved to compute GMI and to find the list of group specific genes for each level of discrimination.

For easy understanding, Fig. 4 depicts the steps involved in the computation of GMI, which are explained next.

#### Normalization

The gene expression values of each gene are normalized in the range from 0 to 1 across samples. This step preserves the richness in the original gene expression values for each gene among the samples and helps us to easily visualize the distribution of gene expression values for the discriminatory genes.

#### Computation of the mean value of each class for each gene

For each gene, the mean of the gene expression values in each class is calculated. Let the mean for gene *g* in class *k* be *μ_gk_*.

#### Sorting of the mean values and defining of K-1 levels of discrimination

For notational simplicity, to explain the computation of the GMI for gene *g*, we ignore the index *g*. We sort *μ_k_*, *k* = 1, 2, …, *K* (*K*  =  number of classes) in descending order. Let the sorted mean values be *μ_k_*_(*s*)_; *k* = 1, 2, …, *K.* Suppose 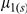 is the mean for class C*_1_*_(*s*)_. This means that the gene under consideration is the most highly expressed in class C*_1_*_(*s*)_. We shall call a gene level-*n* discriminatory gene, if it can discriminate between two groups of classes where one group has *n* classes in it and the other group has the remaining *(K-n*) classes. Moreover, for a level-*n* discriminatory gene, it is highly expressed in the group with *n* classes while in the remaining *K*-*n* classes, this gene is poorly expressed or unexpressed. Note that, if there are *K* classes, we can define (*K-1*) levels of discrimination.

#### Computation of GMI for each level of discrimination

For a good marker gene with clear level-*n* discrimination, there are *n* classes that have high gene expression values and all other classes have low gene expression values. Therefore, we define the set of classes with high gene expression values as “upper group” and the set of remaining classes as “lower group”. For a good marker gene with level-*n* discrimination the upper and lower groups should be well separated and the overlap between the groups should be low. For level-*n* discrimination for gene *g*, first we focus on the separation between upper and lower groups. We use the difference between the mean values of classes C*_n_*_(*s*)_ and C*_n_*_+1(*s*)_, i.e., μ*_Sep_*_  = _ μ*_n_*_(*s*)_ – μ*_n_*_+1(*s*)_. This is the closest separation between a class in the upper group and a class in the lower group. We take this as the separation between the two groups of classes. Note that, we are not using the group mean. Next we define a measure of overlap. For this we find the number of BCTs between these two groups. To consider the effect of sample size, we use a weight parameter, *N_S_*, to normalize the BCT value. Suppose *N*_up_ and *N*_low_ represent the total number of samples in upper and lower groups and let *N_S_*  =  *Min*{*N*_up_, *N*_low_}. Then a measure of overlap between the two groups can be defined as 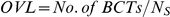. The GMI value for level-*n* discrimination for gene *g* is then defined as the ratio of μ*_Sep_* and *OVL* :
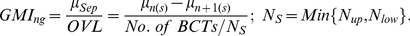
(1)A high value of GMI will indicate good separation with low overlap between the two groups.

#### Finding a list of group specific genes for each level of discrimination

After calculating the GMI values of *K*-1 levels of discrimination for all genes, a list of *group specific genes* for each level can be obtained as follows. Every gene has *K*-1 GMI values. Clearly, genes with higher GMI*_n_* values have better level-*n* discriminating power between upper and lower groups. Now for a given discrimination level-*n,* we sort all genes in descending order of GMI*_n_* values. The sorted values may be denoted as GMI*_n(s)_*. A smaller rank indicates a larger GMI*_n_* value and hence a better level-*n* discriminating power of the gene. From the top of the list of sorted level-*n* genes, we can select a set of genes that is likely to be biologically interesting and is expected to be useful for level-*n* discrimination. Note that, the upper and lower groups associated with different level-*n* genes could be completely different. At this point, we would like to emphasize that designing of classifier or diagnostic prediction system is not the primary objective of this study. The main objective is to find genes that can discriminate between two sets of diseases, not necessarily one disease verses other. We also emphasize that the proposed scheme neither uses pooled mean of a group of classes nor uses the pooled standard deviation of a set of classes and hence is free from the problems of SNR type indexes [1].

### Illustration of GMI Computation

**Figure 5:**
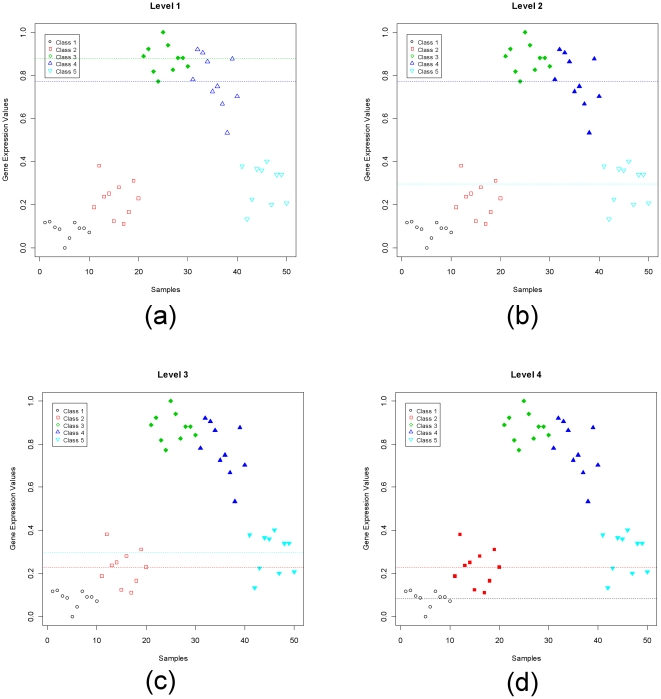
A 5-class synthetic example to illustrate computation of GMI. There are four levels of discrimination in the 5-class synthetic data set. Panels (a) to (d) depict the computation of GMI values at each level of discrimination. The dotted lines in each panel indicate the two mean values used for GMI computation in each level of discrimination. All filled samples in each panel indicate the upper group samples. The remaining open samples in each panel indicate the lower group samples.

To understand the computation of the GMI value at each level of discrimination for a given gene, we have generated a synthetic example, which is composed of five classes of samples. As shown in Fig. 5, there are five classes of samples and there are ten samples in each class. The samples from different classes are labeled with different colors. Since there are *K* = 5 classes, we evaluate the gene for 4 possible levels of discrimination. In Fig. 5, panel *a* to panel *d*, represent, respectively, the computation of level-1 to level-4 discrimination of this gene. In panel *a*, the level-1 discrimination is evaluated between the third class and the fourth class because they are ranked first and second based on the descending order of the mean of the gene expression values in the five classes. These two highest ranked means are used to calculate μ*_Sep_* and this separation is indicated by two horizontal dashed lines in green and blue. For an easy computation of BCTs, in all four panels, the upper and lower groups are represented using filled in and empty symbols, respectively. Similarly, for panels *b* to *d*. For example, in panel *b*, for level-2 discrimination, μ*_Sep_* is computed using the mean values of class four and class five. From these figures, it is clear that this synthetic gene is a good level-2 gene because from panel *b* we find that two groups are well separated and the BCT is just 1. This is also revealed by the GMI values of 0.150, 9.537, 0.287, and 1.925 for level-1 to level-4, respectively.

### Gene selection and evaluation of statistical significance

To evaluate the statistical significance of group specific genes identified by GMI, we perform a permutation procedure to obtain the corresponding *p*- and *q*-values. This procedure is similar to the method used in our previous study [1]. The necessary steps are summarized below. Let *G* be the total number of genes and *S* be the total number of samples.

#### Step 1. Gene selection

Step 1.1 Repeated random splitting. Given a microarray data set *D* with *K* classes (*x_gs_* is the gene expression value of gene *g* in sample *s*; 1≤*g* ≤*G*, 1≤*s*≤*S*) and with class labels (*γ_s_*, 1≤*s*≤*S*), we randomly select 2/3rd samples from each class as the training set *TR_(r)_* (*r* denotes the *r* ^th^ random selection of samples, 1≤*r*≤*R*).

Step 1.2 Computation of GMI and preliminary gene selection. For each training set *TR_(r)_*, we compute the GMI values for different levels of discrimination GMI*_ng(r)_* (1≤*n*≤*K-1*) for each gene *g.* Simultaneously, we use an independent indicator F*_ng(r)_* to denote whether the gene *g* is included in the list of top *N_1_* genes (*N_1_* genes ranked in descending order of GMI*_ng(r)_* values); F*_ng(r)_* = 1 represents “true” and F*_ng(r)_* = 0 represents “false”.

Step 1.3 Gene selection. After *R* = 100 times of the random selection of samples, we average the GMI values of different levels of discrimination, GMI*_ng(r)_*, 1≤*r*≤*R*, for each gene *g* as GMI*_ng(ave)_* and sum up the F*_ng(r)_* values for each gene *g* as F*_ng(sum)_*. Note that, F*_ng(sum)_* is the number of times (i.e., frequency) the gene *g* is selected as one of the top *N_1_* genes in *R* experiments. For each level of discrimination, we select the top *N_2_* genes with the highest frequencies, F*_ng(sum)_*. In this study, we set both *N_1_* and *N_2_* = 10.

#### Step 2. Permutation

We randomly permute the class labels *γ_s_* for *B* times. In the *b* ^th^ permutation (1≤*b*≤*B*), we randomly select 2/3rd samples from each class as the training set *TR_(r)_^(b)^* for *R* = 100 times. For each training set *TR_(r)_^(b)^*, we compute GMI*_ng(r)_* ^(*b*)^ for different levels of discrimination for gene *g* using the permuted class labels *γ_s_* ^(*b*)^. Next, we average these new GMI values of different levels of discrimination GMI*_ng(r)_^(b)^* for each gene *g* as GMI*_ng(ave)_^(b)^*. These GMI*_ng(ave)_^(b)^* are used for the calculation of *p*- and *q*-values.

#### Step 3. Calculation of p-values

The *p*-value of the observed averaged GMI value, GMI*_ng(ave)_*, for a particular level of discrimination, of a gene *g* is
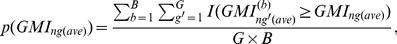
(2)Where *I*(.) is an indicator function that takes the value one when true, and zero otherwise.

#### Step 4. Calculation of q-values

To account for the multiple tests being performed in the *G* genes, the *q*-value of the observed averaged GMI*_ng(ave)_* is calculated as
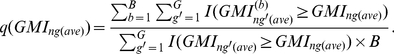
(3)In this study, we have performed this permutation test with *B* = 200 for all data sets.

### Comparison with other method

In order to demonstrate the effectiveness of our GMI method in identifying group specific genes, we propose a scheme based on the template-based method (TBM), which is similar to the method used in a previous work [19] for comparison. In this study, we used Pearson's correlation coefficient to evaluate the relation between gene expression values and pre-assigned templates. The detailed steps of gene selection in the proposed TBM are described below:

#### TBM Step 1: Repeated random splitting

For a fair comparison with GMI, the repeated random splitting scheme is also used here. For a given microarray data set *D* with *K* classes (*x_gs_* is the gene expression value of gene *g* in sample *s*; 1≤*g*≤*G*, 1≤*s*≤*S*) with class labels (*γ_s_*, 1≤*s*≤*S*), we randomly select 2/3rd samples from each class as the training set *TR_(r)_* (as earlier, *r* denotes the *r* ^th^ random selection of samples, 1≤*r*≤*R*).

#### TBM Step 2: Normalization

For each training set *TR_(r)_*, the gene expression values of every gene are normalized across samples to [0, 1].

#### TBM Step 3: Identification of group specific genes for level-n discrimination

For every gene in the training set *TR_(r)_*, *K*-1 levels of discrimination are defined as in the previous section. To find group specific marker genes for level-*n* discrimination, the following steps are followed:

TBM Step 3.1 Creation of template T*_0(r)._*We create a template T*_0(r)_*{0, 0, 0, … , 0}; the length of T*_0(r)_* is equal to the number of samples in training set *TR_(r)_*. Initially, every value in T*_0(r)_* is set to zero.

TBM Step 3.2 Creation of template T*_n(m)._* For a given level-*n*, there could be many combinations (subsets) of classes for the upper group. We denote the appropriate template for the *m* ^th^ combination (for level-*n* discrimination) as T*_n(m)(r)_* and generate it from T*_0(r)_* as follows. If the *s* ^th^ sample of gene *g* belongs to the upper group (i.e., in the *m* ^th^ combination of classes) then the *s* ^th^ value of T*_0(r)_* is set to 1. This modified T*_0(r)_* is the template T*_n(m)(r)_*.

TBM Step 3.3 Computation of Pearson's correlation coefficient. Note that, for level-*n*, *m* ^th^ combination, and sample set *TR_(r)_*, the T*_n(m)(r)_* is fixed. Now we compute the Pearson's correlation coefficient for every gene *g*, with the template T*_n(m)(r)_* and denote it as P*_n(m)(r)_*(*g*). For level-*n* we have 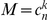 combinations. Let 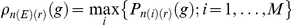. Now we sort the *G* correlation values 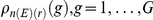 in descending order and create the list of top *N_1_* genes (*along with the associated upper groups*) for level-*n* as L*_n_*_(*r*)_. Thus, a typical element of the list will have two components: the upper group (*E*) and the gene (*g*).

#### TBM Step 4: Gene selection

Steps 1 through 3 are repeated for *r* = 1 to *R* times (here *R* = 100) resulting in *R* lists of top *N_1_* genes, L*_n(r)_*; *r* = 1, …, *R*. Let F*_n_*(*g*, *m*) be the number of times (frequency) with which the gene *g*, associated with upper group *m*, appears in the *R* lists. This results in a list where every gene has associated with it just one frequency and one upper group. Now, we sort this list in descending order based on the frequencies and select the top *N_2_* genes from that list. Note that, each of these *N_2_* genes will have an associated upper group. Here *N_1_* = *N_2_* = 10 as used with GMI.

## Supporting Information

Figure S1 Scatter-plots of the top most gene of each level in the Leukemia data set.
(PDF)

Figure S2 Scatter-plots of the top most gene of each level in the CNS data set.
(PDF)

Figure S3 Scatter-plots of the top most gene of each level in the Lung Cancer data set.
(PDF)

Figure S4 The modified figure of B cell receptor signaling pathway.
(PDF)

Figure S5 The distributions of ANOVA *p*-values for four data sets.
(PDF)

Figure S6 Illustration of between-class-transition (BCT).
(PDF)

Table S1 Summary of top 10 genes of each level selected by GMI in the Leukemia data set.
(PDF)

Table S2 Summary of top 10 genes of each level selected by GMI in the CNS data set.
(PDF)

Table S3 Summary of top 10 genes of each level selected by GMI in the Lung Cancer data set.
(PDF)

Table S4 The comparison of top 10 level-2 genes selected by GMI and TBM in the CNS data set.
(PDF)

Table S5 The comparison of top 10 level-3 genes selected by GMI and TBM in the CNS data set.
(PDF)

Table S6 The comparison of top 10 level-2 genes selected by GMI and TBM in the Leukemia data set.
(PDF)

Table S7 The comparison of top 10 level-2 genes selected by GMI and TBM in the Lung *Cancer data set.*
(PDF)

Table S8 The comparison of top 10 level-3 genes selected by GMI and TBM in the Lung Cancer data set.
(PDF)

Table S9 The full summary table of the identified pathways related to the level 2 discriminatory genes in the Leukemia data set.
(PDF)

Table S10 The full summary table of the identified pathways related to the level 2 discriminatory genes in the Lung cancer data set (Part I).
(PDF)

Table S11 The full summary table of the identified pathways related to the level 2 discriminatory genes in the Lung cancer data set (Part II).
(PDF)

File S1 The level-2 genes selected only by GMI and the level-2 genes selected only by TBM in the Lung Cancer data set.
(PDF)
