# Supplementary material for: Identification of Single- and Multiple-Class Specific Signature Genes from Gene Expression Profiles by Group Marker Index
Source: PLoS One. 2011 Sep 1;6(9):e24259. doi: 10.1371/journal.pone.0024259 (PMC3164723; doi:10.1371/journal.pone.0024259)
Supplement: Figure S1 — Scatter-plots of the top most gene of each level in the Leukemia data set. (PDF) [file pone.0024259.s001.pdf]

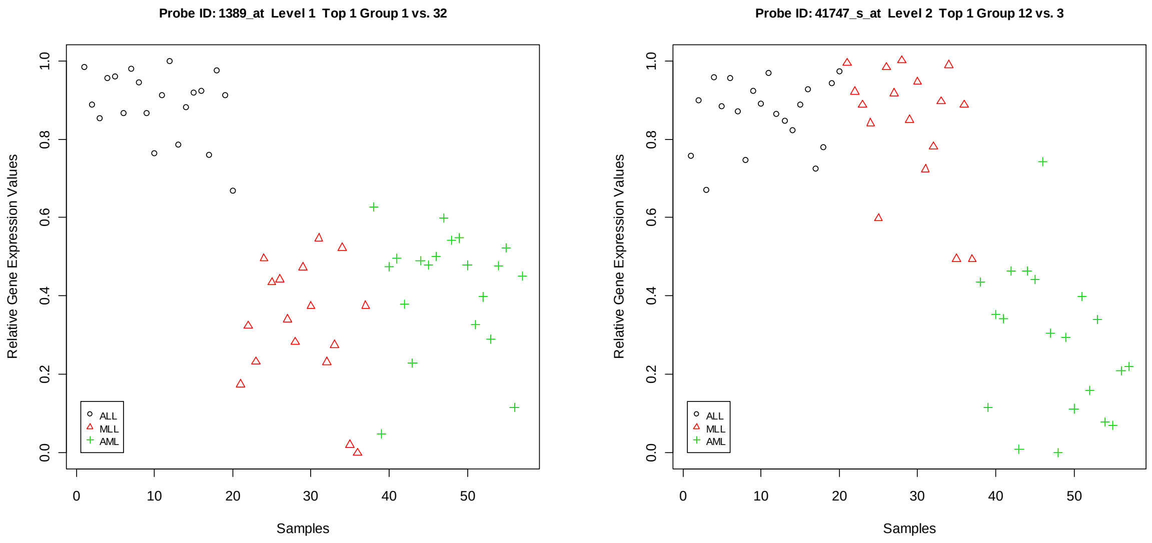

(a) (b)

Figure S1. Scatter-plots of the top most gene of each level in the Leukemia data set. Panels (a) and (b) are the scatter-plots of the top most gene of level-1 and level-2. The top most genes are MME (1389_at) and MEF2A (41747_s_at), respectively. There are three classes in the Leukemia data set: acute lymphoblastic leukemia (ALL), mixed-lineage leukemia (MLL), and acute myelogenous leukemia (AML).
