# Supplementary material for: Identification of Single- and Multiple-Class Specific Signature Genes from Gene Expression Profiles by Group Marker Index
Source: PLoS One. 2011 Sep 1;6(9):e24259. doi: 10.1371/journal.pone.0024259 (PMC3164723; doi:10.1371/journal.pone.0024259)
Supplement: Figure S2 — Scatter-plots of the top most gene of each level in the CNS data set. (PDF) [file pone.0024259.s002.pdf]

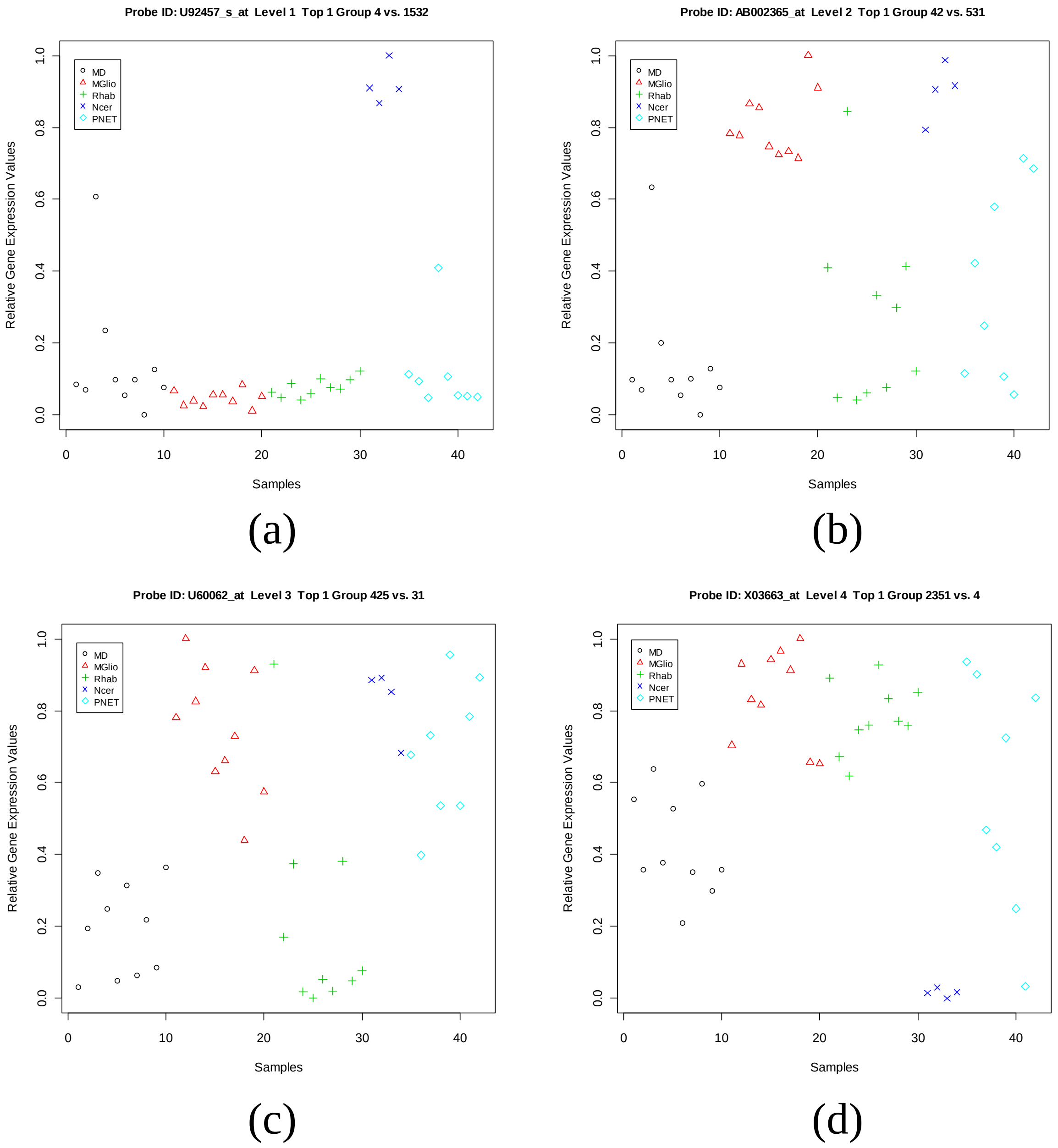

Figure S2. Scatter-plots of the top most gene of each level in the CNS data set. Panels (a), (b), (c) and (d) are the scatter-plots of the top most gene of level-1, level-2, level-3 and level-4, respectively. The top most genes are GRM4 (U92457_s_at), PRUNE2 (AB002365_at), FEZ1 (U60062_at) and GNAI2 (X04828_at), respectively. There are five classes in the CNS data set: medulloblastomas (MD), malignant gliomas (MGlio), atypical teratoid/rhabdoid tumors (Rhab), human cerebella tumors (Ncer), and primitive neuro-ectodermal tumors (PNET).
