# Supplementary material for: Identification of Single- and Multiple-Class Specific Signature Genes from Gene Expression Profiles by Group Marker Index
Source: PLoS One. 2011 Sep 1;6(9):e24259. doi: 10.1371/journal.pone.0024259 (PMC3164723; doi:10.1371/journal.pone.0024259)
Supplement: Figure S3 — Scatter-plots of the top most gene of each level in the Lung Cancer data set. (PDF) [file pone.0024259.s003.pdf]

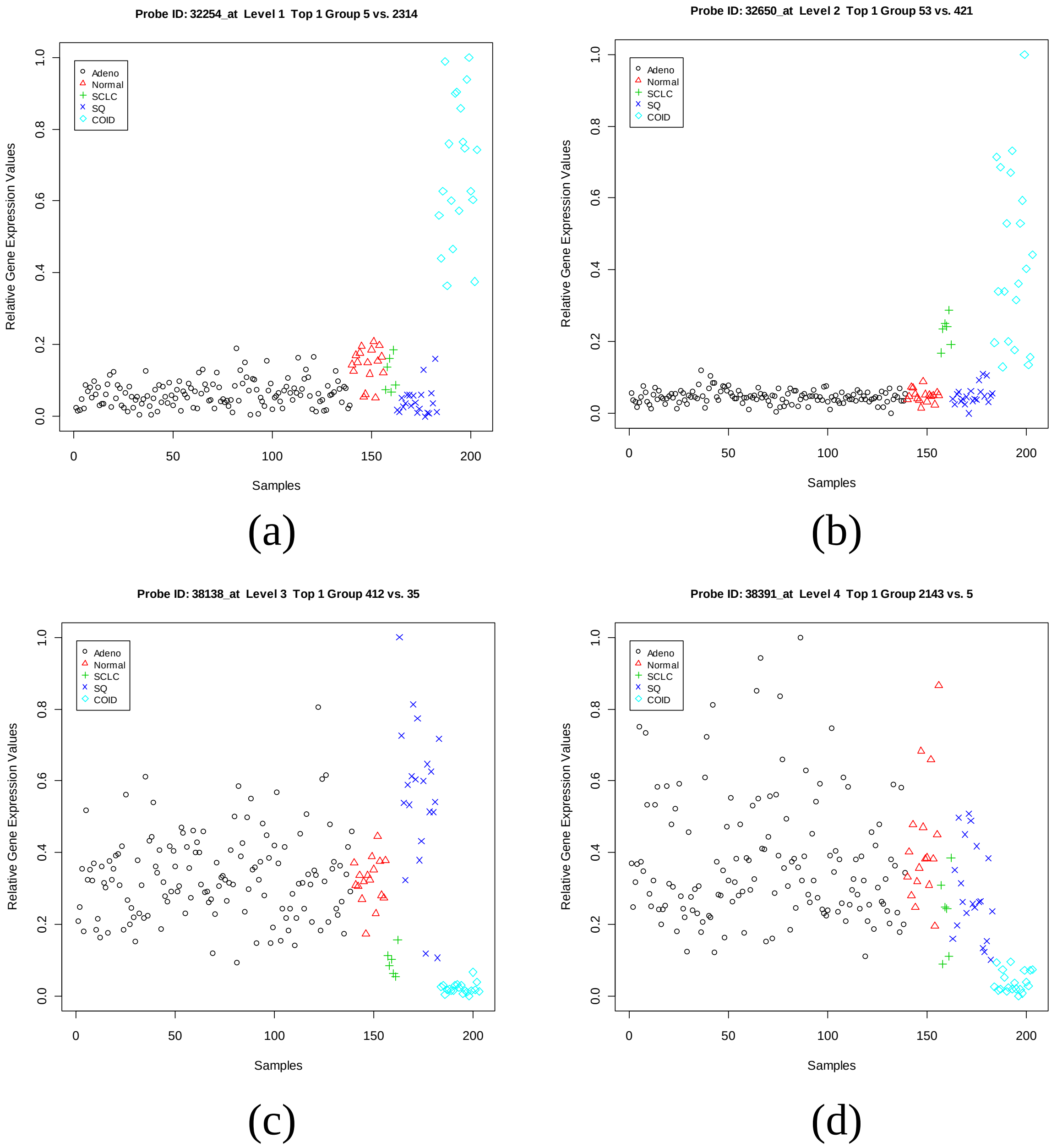

Figure S3. Scatter-plots of the top most gene of each level in the Lung Cancer data set. Panels (a), (b), (c) and (d) are the scatter-plots of the top most gene of level-1, level-2, level-3 and level-4, respectively. The top most genes are VAMP2 (32254_at), TAGLN3 (32650_at), S100A11 (38138_at) and CAPG (38391_at), respectively. There are five classes in the lung cancer data set: lung adenocarcinomas (Adeno), normal lung specimens (Normal), small-cell lung cancer (SCLC), squamous cell lung carcinomas (SQ), and pulmonary carcinoids (COID).
