# Supplementary material for: Identification of Single- and Multiple-Class Specific Signature Genes from Gene Expression Profiles by Group Marker Index
Source: PLoS One. 2011 Sep 1;6(9):e24259. doi: 10.1371/journal.pone.0024259 (PMC3164723; doi:10.1371/journal.pone.0024259)
Supplement: Figure S4 — The modified figure of B cell receptor signaling pathway. (PDF) [file pone.0024259.s004.pdf]

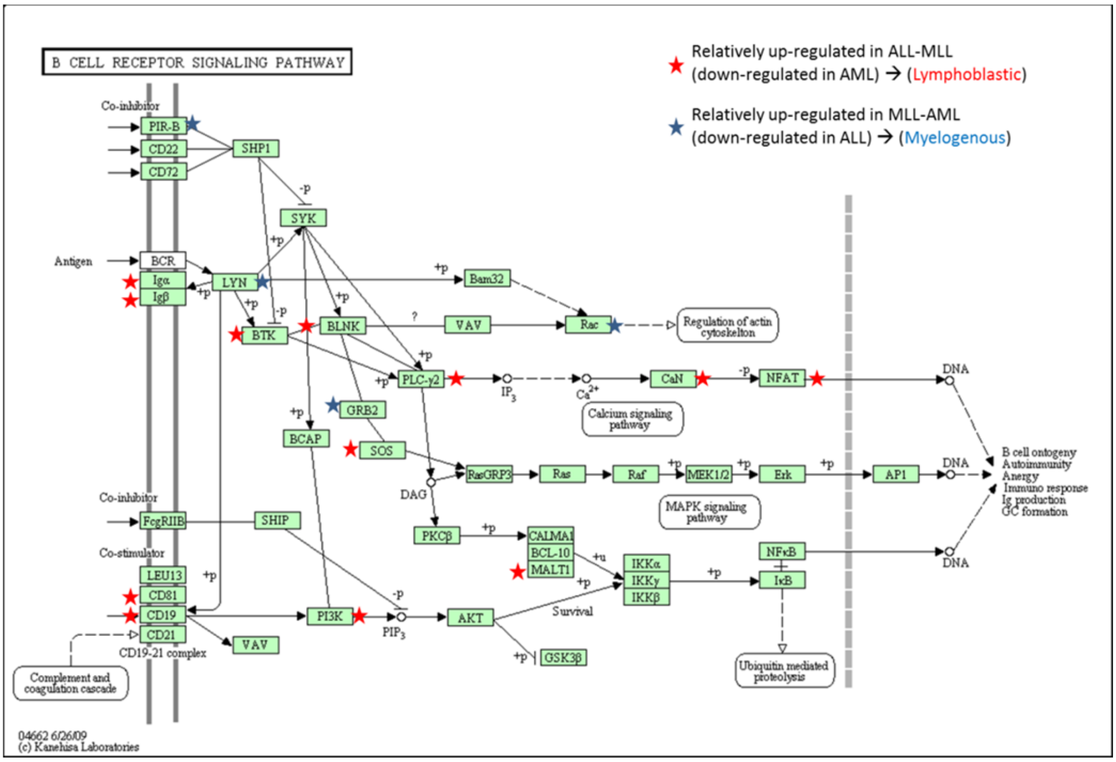

Figure S4. The modified figure of B cell receptor signaling pathway.

As mentioned in the manuscript, those genes, which are relatively up-regulated in the ALL-MLL group, are labeled with red stars. On the other hand, those genes, which are up-regulated in MLL-AML group, are labeled with blue stars. We may interpret those red star genes as lymphoblastic genes because of the relatively up-regulated expression in both ALL and MLL but down-regulated expression in AML. Subsequently, those blue star genes can be treated as myelogenous genes because of their relatively lower expression in ALL. From this figure, we may interpret that the activation of *CD79A* (Igα) and *CD79B* (Igβ) with the co-simulators *CD81* and *CD19* triggers the activation of B cell signaling pathway in ALL and MLL. Furthermore, the co-inhibitor *LIRB3* (PIR-B) is also relatively down-regulated in ALL which could be treated as a positive factor for activating the B cell signaling pathway. Notably, the relatively lower expression of *Rac* in ALL may play an important role of lymphoblastic leukemia because of its function of appropriate positioning of hematopoietic stem cells (HSCs) within the bone marrow microenvironment.

Abbreviation: acute lymphoblastic leukemia (ALL), mixed-lineage leukemia (MLL), and acute myelogenous leukemia (AML).
