# Supplementary figures and images for: Identification of Single- and Multiple-Class Specific Signature Genes from Gene Expression Profiles by Group Marker Index
Source: PLoS One. 2011 Sep 1;6(9):e24259. doi: 10.1371/journal.pone.0024259 (PMC3164723; doi:10.1371/journal.pone.0024259)

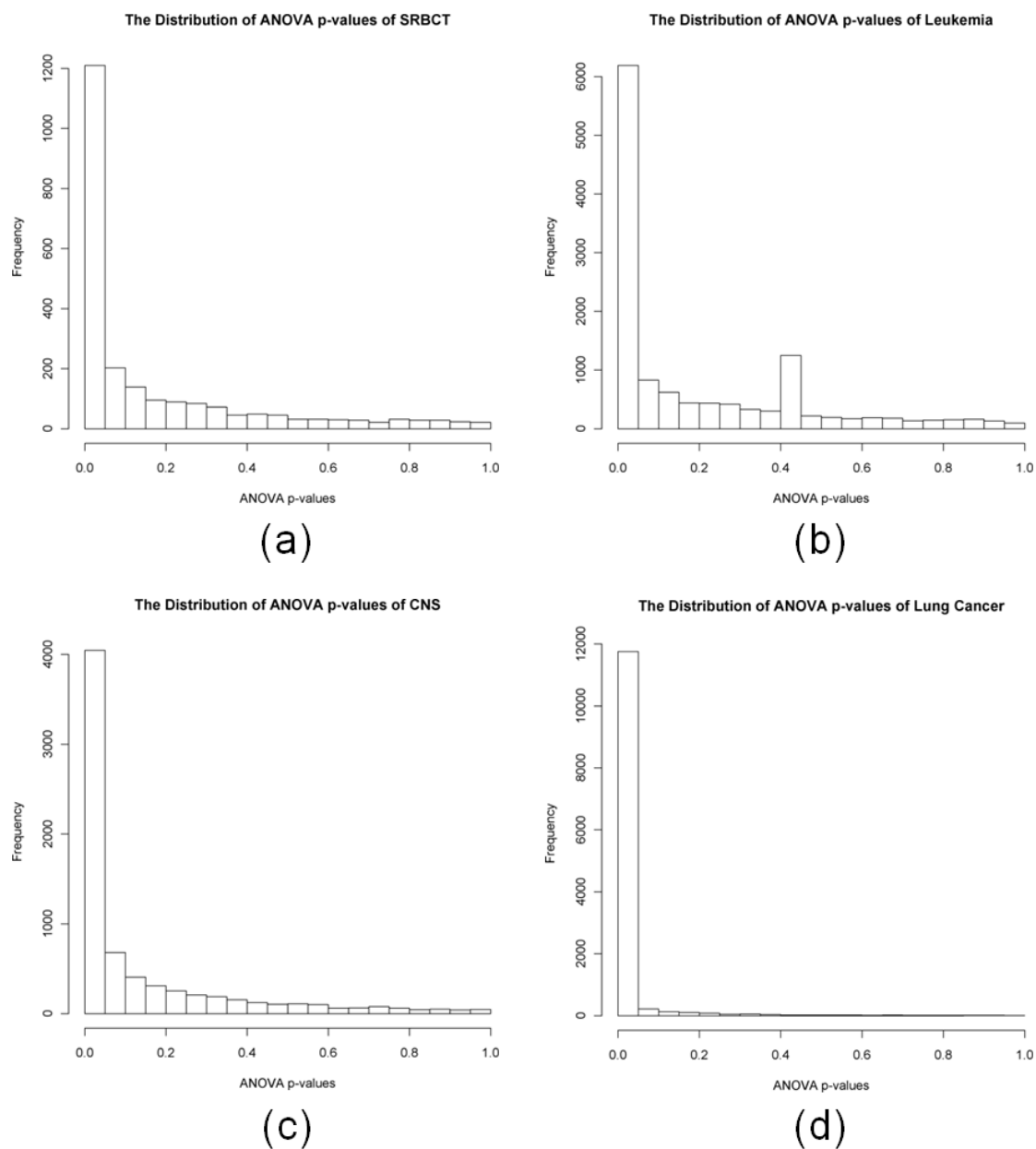

**Figure S5.** The distributions of ANOVA p-values for four datasets.

Supplement: Figure S5 — The distributions of ANOVA p-values for four data sets. (PDF) [file pone.0024259.s005.pdf]
