# Supplementary material for: Identification of Single- and Multiple-Class Specific Signature Genes from Gene Expression Profiles by Group Marker Index
Source: PLoS One. 2011 Sep 1;6(9):e24259. doi: 10.1371/journal.pone.0024259 (PMC3164723; doi:10.1371/journal.pone.0024259)
Supplement: Figure S6 — Illustration of between-class-transition (BCT). (PDF) [file pone.0024259.s006.pdf]

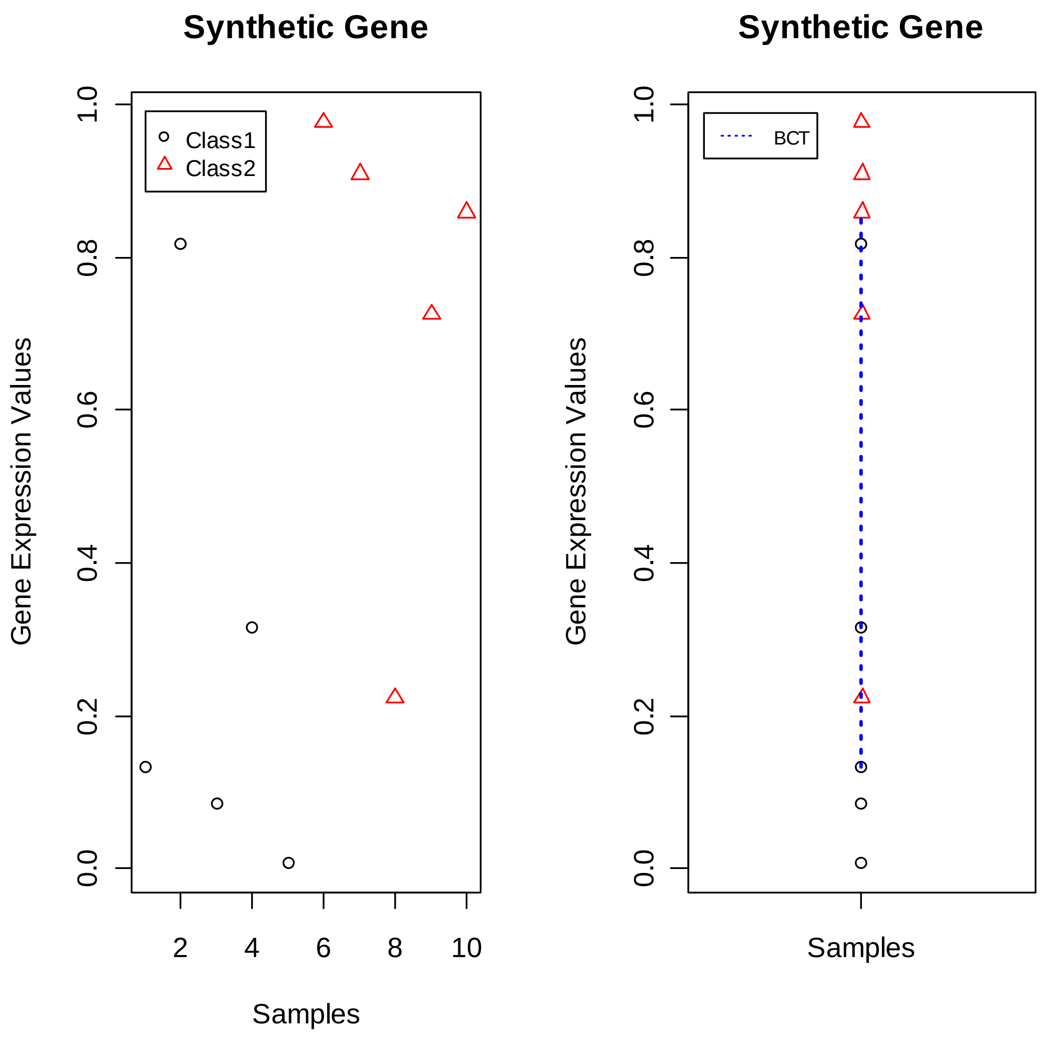

Figure S6. Illustration of between-class-transition (BCT). The left panel shows the scatter-plot of the synthetic gene. After projecting all samples of the synthetic gene on to a line, the samples in the order of gene expression value are shown in the right panel of the figure. The samples involved in the BCT are indicated by the blue dotted line.
