# Supplementary material for: Identification of Single- and Multiple-Class Specific Signature Genes from Gene Expression Profiles by Group Marker Index
Source: PLoS One. 2011 Sep 1;6(9):e24259. doi: 10.1371/journal.pone.0024259 (PMC3164723; doi:10.1371/journal.pone.0024259)
Supplement: Table S1 — Summary of top 10 genes of each level selected by GMI in the Leukemia data set. (PDF) [file pone.0024259.s007.pdf]

Table S1. Summary of top 10 genes of each level selected by GMI in the Leukemia data set.

Level	Probe ID	Gene Symbol	Upper Group	Lower Group	Freq.	Ave. GMI Value	p-value	q-value
1	1389_at	MME	1	32	100	6.09	0	0
	35260_at	MLXIP	1	23	85	3.63	0	0
	32847_at	MYLK	1	32	72	2.97	0	0
	37280_at	SMAD1	1	32	44	2.86	0	0
	34168_at	DNTT	1	23	41	2.49	0	0
	39566_at	CHRNA7	3	21	41	2.76	0	0
	36239_at	POU2AF1	1	23	37	3.00	0	0
	40763_at	MEIS1	2	31	37	2.83	0	0
	35164_at	WFS1	1	23	29	2.05	0	0
	32872_at	TCF4	1	23	25	2.30	0	0
2	41747_s_at	MEF2A	12	3	99	4.24	0	0
	33412_at	LGALS1	23	1	66	3.21	0	0
	41503_at	ZHX2	12	3	47	2.82	0	0
	37710_at	MEF2C	12	3	44	2.14	0	0
	40966_at	STK39	12	3	44	2.64	0	0
	40701_at	USP13	12	3	41	2.03	0	0
	37403_at	ANXA1	32	1	41	2.42	0	0
	37535_at	CREB1	12	3	40	2.42	0	0
	38242_at	BLNK	12	3	28	1.86	0	0
	37561_at	NFYA	12	3	26	1.89	0	0

Acute lymphoblastic leukemia (ALL), mixed-lineage leukemia (MLL), and acute myelogenous leukemia (AML) are represented as Group 1 to Group 3 in order.
