# Supplementary material for: Identification of Single- and Multiple-Class Specific Signature Genes from Gene Expression Profiles by Group Marker Index
Source: PLoS One. 2011 Sep 1;6(9):e24259. doi: 10.1371/journal.pone.0024259 (PMC3164723; doi:10.1371/journal.pone.0024259)
Supplement: Table S2 — Summary of top 10 genes of each level selected by GMI in the CNS data set. (PDF) [file pone.0024259.s008.pdf]

Table S2. Summary of top 10 genes of each level selected by GMI in the CNS data set.

Level	Probe ID	Gene Symbol	Upper Group	Lower Group	Freq.	Ave. GMI Value	p-value	q-value
1	U92457_s_at	GRM4	4	1532	78	2.37	0	0
	D87463_at	PHYHIP	4	2153	60	2.22	0	0
	J04164_at	IFITM1	3	5412	47	2.20	0	0
	X63578_rna1_at	PVALB	4	5132	41	2.14	0	0
	D26070_at	ITPR1	4	2153	40	2.16	0	0
	D84454_at	SLC35A2	3	1524	38	2.48	0	0
	L38969_at	THBS3	3	1452	36	2.23	0	0
	U79245_at	SLC12A5	4	1532	35	1.58	0	0
	L02950_at	CRYM	4	1235	23	2.04	0	0
	HG2259-HT2348_s_at	TUBA4A	4	5132	23	1.22	0	0
2	AB002365_at	PRUNE2	42	531	90	2.38	0	0
	U76456_at	TIMP4	42	153	59	1.68	0	0
	M77016_at	TMOD1	42	135	58	1.75	0	0
	L22214_at	ADORA1	24	513	50	1.65	0	0
	D82347_at	NEUROD1	41	523	38	1.26	0	0
	Z29505_at	PCBP1	13	524	28	1.22	0	0
	U49857_at	C1orf61	42	513	26	1.17	0	0
	HG662-HT662_at	RPL22P11	13	524	20	1.01	0	0
	M63623_at	OMG	24	513	20	0.99	0	0
	S76475_at	NTRK3	41	253	15	0.83	7.01E-07	7.01E-07
3	U60062_at	FEZ1	425	31	100	3.95	0	0
	L76159_at	FRG1	143	52	75	2.07	0	0
	X13546_rna1_at	HMGN2	135	42	32	1.11	0	0
	U18009_at	VAT1	312	54	30	1.14	0	0
	X63359_at	ADH6	452	13	30	0.97	0	0
	X16560_at	COX7C	314	52	24	0.95	0	0
	AF002224_at	UBE3A	425	13	20	0.79	7.01E-07	7.01E-07
	HG1602-HT1602_at	utrn	452	13	20	0.83	7.01E-07	7.01E-07
	D21267_at	SNAP25	412	53	19	0.74	1.40E-06	1.40E-06
	M31303_rna1_at	STMN1	451	32	19	0.88	7.01E-07	7.01E-07
4	U52828_s_at	CTNND2	2415	3	44	1.89	0	0
	X02761_s_at	FN1	3521	4	43	1.16	0	0
	Y07829_xpt4_at	TRIM15	2451	3	35	1.76	0	0

X04828_at	GNAI2	2351	4	34	1.24	0	0
HG3546-HT3744_s_at	SFRS1	1352	4	27	0.74	7.01E-07	7.01E-07
HG2825-HT2949_at	TRIM27	2153	4	25	0.86	7.01E-07	7.01E-07
U83239_s_at	CCL22	5241	3	25	0.95	7.01E-07	7.01E-07
D59253_at	NCBP2	1325	4	21	0.76	7.01E-07	7.01E-07
M21812_at	MYLPF	4251	3	21	0.96	0	0
M55905_at	ME2	3152	4	20	0.60	8.42E-06	8.42E-06

Medulloblastomas (MD), malignant gliomas (MGlio), atypical teratoid/rhabdoid tumors (Rhab), human cerebella tumors (Ncer), and primitive neuro-ectodermal tumors (PNET) are represented as Group 1 to Group 5 in order.
