# Supplementary material for: Identification of Single- and Multiple-Class Specific Signature Genes from Gene Expression Profiles by Group Marker Index
Source: PLoS One. 2011 Sep 1;6(9):e24259. doi: 10.1371/journal.pone.0024259 (PMC3164723; doi:10.1371/journal.pone.0024259)
Supplement: Table S3 — Summary of top 10 genes of each level selected by GMI in the Lung Cancer data set. (PDF) [file pone.0024259.s009.pdf]

Table S3. Summary of top 10 genes of each level selected by GMI in the Lung Cancer data set.

Level	Probe ID	Gene Symbol	Upper Group	Lower Group	Freq.	Ave. GMI Value	p-value	q-value
1	32254_at	VAMP2	5	2314	100	7.19	0	0
	41675_at	SNAP91	5	3142	98	6.86	0	0
	40808_at	CHGA	5	3142	95	6.89	0	0
	40825_at	MAPRE3	5	3241	83	6.77	0	0
	40165_at	TSPYL2	5	3214	73	6.32	0	0
	41107_at	SNPH	5	3124	70	6.18	0	0
	916_at	PTPRN	5	3124	67	6.15	0	0
	36148_at	APLP1	5	3124	36	5.44	0	0
	35592_at	SLC8A2	5	3124	26	4.15	0	0
	34847_s_at	CAMK2B	5	3142	23	2.98	0	0
2	32650_at	TAGLN3	53	421	100	3.45	0	0
	40272_at	CRMP1	53	412	100	2.94	0	0
	41289_at	NCAM1	53	124	93	2.53	0	0
	33157_at	INSM1	35	142	70	1.58	0	0
	37545_at	SCAMP5	53	142	61	1.56	0	0
	35778_at	KIF5C	53	142	44	1.36	0	0
	38146_at	ST18	53	124	40	1.17	0	0
	38163_at	RIMS2	53	142	39	1.18	0	0
	39666_at	GNG4	35	142	38	0.96	0	0
	41338_at	AES	53	241	37	1.26	0	0
3	38138_at	S100A11	412	35	100	1.25	0	0
	32715_at	VAMP8	214	35	100	0.88	0	0
	36207_at	SEC14L1	532	41	99	0.55	0	0
	35367_at	LGALS3	214	35	84	0.50	0	0
	39338_at	S100A10	214	35	54	0.35	0	0
	37281_at	FAM38A	214	35	50	0.34	0	0
	38368_at	DUT	534	21	46	0.33	0	0
	41222_at	STAT6	214	35	32	0.29	0	0
	40203_at	EIF1	352	41	28	0.28	0	0
	38084_at	CBX3	341	52	27	0.30	0	0
4	38391_at	CAPG	2143	5	99	1.44	0	0
	925_at	IFI30	2134	5	87	0.81	0	0
	39728_at	IFI30	2134	5	82	0.84	0	0

769_s_at	ANXA2	4213	5	79	0.72	0	0
31444_s_at	ANXA2P3	4213	5	73	0.65	0	0
37024_at	LITAF	2143	5	62	0.66	0	0
31481_s_at	TMSB10	1423	5	60	0.61	0	0
33390_at	CD68	2134	5	47	0.64	0	0
38194_s_at	IGKV1-5	3142	5	42	0.52	0	0
38138_at	S100A11	4123	5	32	0.55	0	0

Lung adenocarcinomas (Adeno), normal lung specimens (Normal), small-cell lung cancer (SCLC), squamous cell lung carcinomas (SQ), and pulmonary carcinoids (COID) are represented as Group 1 to Group 5 in order.
