# Supplementary material for: Identification of Single- and Multiple-Class Specific Signature Genes from Gene Expression Profiles by Group Marker Index
Source: PLoS One. 2011 Sep 1;6(9):e24259. doi: 10.1371/journal.pone.0024259 (PMC3164723; doi:10.1371/journal.pone.0024259)
Supplement: Table S4 — The comparison of top 10 level-2 genes selected by GMI and TBM in the CNS data set. (PDF) [file pone.0024259.s010.pdf]

Table S4. The comparison of top 10 level-2 genes selected by GMI and TBM in the CNS data set.

Probe ID	GMI Mean Order	GMI Rank	GMI Freq.	TBM Rank	TBM Template	TBM Freq.	LOOCV NNC Acc.
AB002365_at	(42)(531)	1	90	4	(24)(135)	60	0.9048
U76456_at	(42)(153)	2	59	2	(24)(135)	83	0.8571
M77016_at	(42)(135)	3	58	1	(24)(135)	93	0.9286
L22214_at	(24)(513)	4	50	6	(24)(135)	31	0.9048
D82347_at	(41)(523)	5	38	3	(14)(235)	71	0.8571
Z29505_at	(13)(524)	6	28	15	(13)(245)	14	0.8333
U49857_at	(42)(513)	7	26	7	(24)(135)	28	0.8810
HG662-HT662_at	(13)(524)	8	20	9	(13)(245)	17	0.8571
M63623_at	(24)(513)	9	20	5	(24)(135)	56	0.7857
S76475_at	(41)(253)	10	15	80	(14)(235)	2	0.9286
X07384_at	(13)(524)	10	15				0.8333
U67156_at	(42)(513)	19	10	8	(24)(135)	24	0.8810
X52882_at	(31)(254)	14	13	10	(13)(245)	17	0.8571

TBM: Template-based method.

Medulloblastomas (MD), malignant gliomas (MGlio), atypical teratoid/rhabdoid tumors (Rhab), human cerebella tumors (Ncer), and primitive neuro-ectodermal tumors (PNET) are represented as Group 1 to Group 5 in order.
