# Supplementary material for: Identification of Single- and Multiple-Class Specific Signature Genes from Gene Expression Profiles by Group Marker Index
Source: PLoS One. 2011 Sep 1;6(9):e24259. doi: 10.1371/journal.pone.0024259 (PMC3164723; doi:10.1371/journal.pone.0024259)
Supplement: Table S5 — The comparison of top 10 level-3 genes selected by GMI and TBM in the CNS data set. (PDF) [file pone.0024259.s011.pdf]

Table S5. The comparison of top 10 level-3 genes selected by GMI and TBM in the CNS data set.

Probe ID	GMI Mean Order	GMI Rank	GMI Freq.	TBM Rank	TBM Template	TBM Freq.	LOOCV NNC Acc.
U60062_at	(425)(31)	1	100	2	(245)(13)	69	0.9286
L76159_at	(143)(52)	2	75	1	(134)(25)	94	0.9048
X13546_rna1_at	(135)(42)	3	32	3	(135)(24)	53	0.9286
U18009_at	(312)(54)	4	30	9	(123)(45)	23	0.9524
X63359_at	(452)(13)	5	30	63	(245)(13)	4	0.8095
X16560_at	(314)(52)	6	24	5	(134)(25)	39	0.8571
AF002224_at	(425)(13)	7	20	58	(245)(13)	4	0.8095
HG1602-HT1602_at	(452)(13)	8	20	39	(245)(13)	6	0.8571
D21267_at	(412)(53)	9	19	16	(124)(35)	11	0.7143
M31303_rna1_at	(451)(32)	10	19	25	(145)(23)	8	0.7619
M14483_rna1_s_at	(135)(24)	12	16	4	(135)(24)	49	0.9048
X74614_at	(245)(13)	45	5	6	(245)(13)	34	0.8095
D86973_at	(135)(42)	41	5	7	(135)(24)	25	0.8095
X86809_at	(245)(13)	20	11	8	(245)(13)	25	0.8810
M21812_at	(425)(13)	54	4	10	(245)(13)	19	0.8333

TBM: Template-based method.

Medulloblastomas (MD), malignant gliomas (MGlio), atypical teratoid/rhabdoid tumors (Rhab), human cerebella tumors (Ncer), and primitive neuro-ectodermal tumors (PNET) are represented as Group 1 to Group 5 in order.
