# Supplementary material for: Identification of Single- and Multiple-Class Specific Signature Genes from Gene Expression Profiles by Group Marker Index
Source: PLoS One. 2011 Sep 1;6(9):e24259. doi: 10.1371/journal.pone.0024259 (PMC3164723; doi:10.1371/journal.pone.0024259)
Supplement: Table S6 — The comparison of top 10 level-2 genes selected by GMI and TBM in the Leukemia data set. (PDF) [file pone.0024259.s012.pdf]

Table S6. The comparison of top 10 level-2 genes selected by GMI and TBM in the Leukemia data set.

Probe ID	GMI Mean Order	GMI Rank	GMI Freq.	TBM Rank	TBM Template	TBM Freq.	LOOCV NNC Acc.
41747_s_at	(12)(3)	1	99	1	(12)(3)	97	0.9649
33412_at	(23)(1)	2	66	3	(23)(1)	81	0.9123
41503_at	(12)(3)	3	47	2	(12)(3)	89	0.8947
37710_at	(12)(3)	4	44	5	(12)(3)	58	0.9298
40966_at	(12)(3)	5	44	14	(12)(3)	18	0.9123
40701_at	(12)(3)	6	41	16	(12)(3)	17	0.8596
37403_at	(32)(1)	7	41	7	(23)(1)	45	0.8947
37535_at	(12)(3)	8	40	4	(12)(3)	64	0.8947
38242_at	(12)(3)	9	28	9	(12)(3)	42	0.9298
37561_at	(12)(3)	10	26	11	(12)(3)	33	0.9123
38017_at	(12)(3)	12	24	6	(12)(3)	56	0.8772
1096_g_at	(12)(3)	12	24	8	(12)(3)	42	0.9474
37988_at	(12)(3)	12	24	10	(12)(3)	40	0.8246

TBM: Template-based method.

Acute lymphoblastic leukemia (ALL), mixed-lineage leukemia (MLL), and acute myelogenous leukemia (AML) are represented as Group 1 to Group 3 in order.
