# Supplementary material for: Identification of Single- and Multiple-Class Specific Signature Genes from Gene Expression Profiles by Group Marker Index
Source: PLoS One. 2011 Sep 1;6(9):e24259. doi: 10.1371/journal.pone.0024259 (PMC3164723; doi:10.1371/journal.pone.0024259)
Supplement: Table S7 — The comparison of top 10 level-2 genes selected by GMI and TBM in the Lung Cancer data set. (PDF) [file pone.0024259.s013.pdf]

Table S7. The comparison of top 10 level-2 genes selected by GMI and TBM in the Lung Cancer data set.

Probe ID	GMI Mean Order	GMI Rank	GMI Freq.	TBM Rank	TBM Template	TBM Freq.	LOOCV NNC Acc.
32650_at	(53)(421)	1	100	23	(35)(124)	8	1.0000
40272_at	(53)(412)	2	100	4	(35)(124)	81	0.9852
41289_at	(53)(124)	3	93	1	(35)(124)	99	0.9655
33157_at	(35)(142)	4	70	11	(35)(124)	33	0.9704
37545_at	(53)(142)	5	61	7	(35)(124)	58	0.9803
35778_at	(53)(142)	6	44	22	(35)(124)	10	0.9606
38146_at	(53)(124)	7	40	36	(35)(124)	3	0.9655
38163_at	(53)(142)	8	39	12	(35)(124)	32	0.9507
39666_at	(35)(142)	9	38	16	(35)(124)	16	0.9557
41338_at	(53)(241)	10	37	14	(35)(124)	19	0.9655
36160_s_at	(53)(124)	25	10	2	(35)(124)	98	0.9458
38032_at	(53)(412)	12	25	3	(35)(124)	87	0.9754
36148_at	(53)(124)	49	2	5	(35)(124)	72	0.9606
34847_s_at	(53)(142)	48	2	6	(35)(124)	64	0.9655
37210_at	(53)(142)	46	2	8	(35)(124)	53	0.9655
38174_at	(53)(142)	36	4	9	(35)(124)	40	0.9754
40421_at	(53)(214)	15	20	10	(35)(124)	39	0.9754

TBM: Template-based method.

Lung adenocarcinomas (Adeno), normal lung specimens (Normal), small-cell lung cancer (SCLC), squamous cell lung carcinomas (SQ), and pulmonary carcinoids (COID) are represented as Group 1 to Group 5 in order.
