# Supplementary material for: Identification of Single- and Multiple-Class Specific Signature Genes from Gene Expression Profiles by Group Marker Index
Source: PLoS One. 2011 Sep 1;6(9):e24259. doi: 10.1371/journal.pone.0024259 (PMC3164723; doi:10.1371/journal.pone.0024259)
Supplement: Table S8 — The comparison of top 10 level-3 genes selected by GMI and TBM in the Lung Cancer data set. (PDF) [file pone.0024259.s014.pdf]

Table S8. The comparison of top 10 level-3 genes selected by GMI and TBM in the Lung Cancer data set.

Probe ID	GMI Mean Order	GMI Rank	GMI Freq.	TBM Rank	TBM Template	TBM Freq.	LOOCV NNC Acc.
38138_at	(412)(35)	1	100				0.9606
32715_at	(214)(35)	2	100				0.9507
36207_at	(532)(41)	3	99	1	(235)(14)	100	0.9015
35367_at	(214)(35)	4	84				0.9458
39338_at	(214)(35)	5	54				0.9064
37281_at	(214)(35)	6	50				0.9310
38368_at	(534)(21)	7	46	7	(345)(12)	66	0.8522
41222_at	(214)(35)	8	32				0.9064
40203_at	(352)(41)	9	28	9	(235)(14)	50	0.8621
38084_at	(341)(52)	10	27				0.8374
41338_at	(532)(41)	37	4	2	(235)(14)	100	0.8966
40202_at	(251)(34)			3	(235)(14)	94	0.7833
1420_s_at	(534)(21)	12	25	4	(345)(12)	92	0.8571
40607_at	(253)(14)			5	(235)(14)	79	0.8916
36894_at	(523)(14)			6	(235)(14)	69	0.8177
41325_at	(523)(14)	45	2	8	(235)(14)	59	0.8867
32780_at	(253)(41)			10	(235)(14)	44	0.8079

TBM: Template-based method.

Lung adenocarcinomas (Adeno), normal lung specimens (Normal), small-cell lung cancer (SCLC), squamous cell lung carcinomas (SQ), and pulmonary carcinoids (COID) are represented as Group 1 to Group 5 in order.
