# Supplementary material for: Identification of Single- and Multiple-Class Specific Signature Genes from Gene Expression Profiles by Group Marker Index
Source: PLoS One. 2011 Sep 1;6(9):e24259. doi: 10.1371/journal.pone.0024259 (PMC3164723; doi:10.1371/journal.pone.0024259)
Supplement: Table S9 — The full summary table of the identified pathways related to the level 2 discriminatory genes in the Leukemia data set. (PDF) [file pone.0024259.s015.pdf]

Table S9. The full summary table of the identified pathways related to the level 2 discriminatory genes in Leukemia data set.

Term	Count	%	PValue	Genes	List Total	Pop Hits	Pop Total	Fold Enrichment	Bonferroni	Benjamini	FDR
ALL-MLL List											
hsa03040:Spliceosome	21	3.1579	5.38E-07	37506_AT, 41224_AT, 37508_F_AT, 38762_AT, 36098_AT, 38031_AT, 41746_AT, 40465_AT, 38654_AT, 41712_AT, 36099_AT, 36991_AT, 33817_AT, 32792_AT, 34325_AT, 34345_AT, 38399_AT, 39896_AT, 32165_AT, 32124_AT, 41836_AT, 32789_AT, 36847_R_AT	228	126	5085	3.7171	8.23E-05	8.23E-05	6.44E-04
hsa04662:B cell receptor signaling pathway	14	2.1053	2.13E-05	38242_AT, 1096_G_AT, 38213_AT, 38017_AT, 32121_AT, 32857_AT, 40822_AT, 1085_S_AT, 37180_AT, 38575_AT, 1116_AT, 40704_AT, 38018_G_AT, 39780_AT, 37988_AT, 35282_R_AT, 32541_AT	228	75	5085	4.1632	0.0033	0.0016	0.0256
hsa03022:Basal transcription factors	7	1.0526	0.0040	37295_AT, 31797_AT, 35450_S_AT, 131_AT, 38782_AT, 802_AT, 41050_AT	228	35	5085	4.4605	0.4623	0.1868	4.7433
hsa00562:Inositol phosphate metabolism	8	1.2030	0.0095	1085_S_AT, 37180_AT, 39323_AT, 41715_AT, 32085_AT, 40704_AT, 35741_AT, 32121_AT, 32133_AT	228	54	5085	3.3041	0.7685	0.3064	10.8272
hsa05210:Colorectal cancer	10	1.5038	0.0120	36021_AT, 1909_AT, 860_AT, 1238_AT, 40704_AT, 36953_AT, 861_G_AT, 32121_AT, 1017_AT, 1814_AT, 2003_S_AT, 32857_AT	228	84	5085	2.6551	0.8413	0.3080	13.4271
hsa03420:Nucleotide excision repair	7	1.0526	0.0126	40141_AT, 1470_AT, 32397_R_AT, 39162_AT, 1054_AT, 1885_AT, 38782_AT	228	44	5085	3.5481	0.8565	0.2765	14.1065
hsa04910:Insulin signaling pathway	13	1.9549	0.0168	32599_AT, 35632_AT, 40139_AT, 38994_AT, 1238_AT, 32121_AT, 32857_AT, 40635_AT, 38813_AT, 33162_AT, 1602_AT, 514_AT, 40704_AT, 1603_G_AT, 37188_AT	228	135	5085	2.1477	0.9248	0.3091	18.3459
hsa03430:Mismatch repair	5	0.7519	0.0175	31884_AT, 860_AT, 1470_AT, 1054_AT, 861_G_AT, 1017_AT, 2003_S_AT	228	23	5085	4.8484	0.9333	0.2871	19.1067
hsa05220:Chronic myeloid leukemia	9	1.3534	0.0178	35632_AT, 514_AT, 2044_S_AT, 943_AT, 40704_AT, 36953_AT, 38443_AT, 32121_AT, 39421_AT, 1814_AT, 32857_AT	228	75	5085	2.6763	0.9363	0.2636	19.3979
hsa03410:Base excision repair	6	0.9023	0.0184	37303_AT, 1470_AT, 40865_AT, 1516_G_AT, 41146_AT, 1287_AT, 38335_AT	228	35	5085	3.8233	0.9419	0.2477	19.9781
hsa05340:Primary immunodeficiency	6	0.9023	0.0184	38242_AT, 1096_G_AT, 38213_AT, 38017_AT, 32144_AT, 1116_AT, 38018_G_AT, 36227_AT, 1370_AT	228	35	5085	3.8233	0.9419	0.2477	19.9781
hsa04660:T cell receptor signaling pathway	11	1.6541	0.0216	33291_AT, 36905_AT, 38575_AT, 35632_AT, 514_AT, 1238_AT, 40704_AT, 39780_AT, 32121_AT, 40822_AT, 32857_AT, 32541_AT	228	108	5085	2.2716	0.9646	0.2620	23.0243
hsa00071:Fatty acid metabolism	6	0.9023	0.0312	37707_I_AT, 37532_AT, 40459_AT, 34790_AT, 33881_AT, 37982_AT	228	40	5085	3.3454	0.9922	0.3327	31.6244
hsa04120:Ubiquitin mediated proteolysis	12	1.8045	0.0414	604_AT, 890_AT, 33136_AT, 35632_AT, 36579_AT, 40623_AT, 1725_S_AT, 40141_AT, 41110_AT, 39723_AT, 38705_AT, 514_AT, 34372_AT	228	137	5085	1.9535	0.9985	0.3923	39.7743
hsa04070:Phosphatidyl inositol signaling system	8	1.2030	0.0459	1085_S_AT, 37180_AT, 39323_AT, 41715_AT, 32085_AT, 40704_AT, 35741_AT, 32121_AT, 32133_AT	228	74	5085	2.4111	0.9992	0.4014	43.0280
MLL-AML List											
hsa05221:Acute myeloid leukemia	4	4.4944	0.0055	1827_S_AT, 32550_R_AT, 1914_AT, 33855_AT	33	58	5085	10.6270	0.3252	0.3252	5.5876
hsa04662:B cell receptor signaling pathway	4	4.4944	0.0112	32737_AT, 37148_AT, 33855_AT, 1402_AT	33	75	5085	8.2182	0.5520	0.3307	11.0767
hsa04664:Fc epsilon RI signaling pathway	4	4.4944	0.0125	36889_AT, 32737_AT, 33855_AT, 1402_AT	33	78	5085	7.9021	0.5909	0.2577	12.2495
hsa04062:Chemokine signaling pathway	5	5.6180	0.0285	32737_AT, 1403_S_AT, 33855_AT, 35372_R_AT, 1402_AT	33	187	5085	4.1201	0.8715	0.4013	25.9129
