# Supplementary material for: Identification of Single- and Multiple-Class Specific Signature Genes from Gene Expression Profiles by Group Marker Index
Source: PLoS One. 2011 Sep 1;6(9):e24259. doi: 10.1371/journal.pone.0024259 (PMC3164723; doi:10.1371/journal.pone.0024259)
Supplement: Table S10 — The full summary table of the identified pathways related to the level 2 discriminatory genes in the Lung cancer data set (Part I). (PDF) [file pone.0024259.s016.pdf]

Table S10. The full summary table of the identified pathways related to the level 2 discriminatory genes in Lung Cancer data set (Part I).

Term	Count	%	PValue	Genes	List Total	Pop Hits	Pop Total	Fold Enrichment	Bonferroni	Benjamini	FDR
Adeno-Normal List											
hsa05416:Viral myocarditis	10	9.0909	1.17E-07	36773_F_AT, 41723_S_AT, 37421_F_AT, 38095_I_AT, 40369_F_AT, 33261_AT, 39695_AT, 40370_F_AT, 37039_AT, 36878_F_AT, 37344_AT, 32640_AT	62	71	5085	11.5516	1.17E-05	1.17E-05	1.30E-04
hsa05330:Allograft rejection	8	7.2727	1.60E-07	36773_F_AT, 41723_S_AT, 37421_F_AT, 38095_I_AT, 40369_F_AT, 33261_AT, 40370_F_AT, 37039_AT, 36878_F_AT, 37344_AT	62	36	5085	18.2258	1.60E-05	7.99E-06	1.77E-04
hsa05332:Graft-versus -host disease	8	7.2727	2.86E-07	36773_F_AT, 41723_S_AT, 37421_F_AT, 38095_I_AT, 40369_F_AT, 33261_AT, 40370_F_AT, 37039_AT, 36878_F_AT, 37344_AT	62	39	5085	16.8238	2.86E-05	9.55E-06	3.18E-04
hsa04940:Type I diabetes mellitus	8	7.2727	4.88E-07	36773_F_AT, 41723_S_AT, 37421_F_AT, 38095_I_AT, 40369_F_AT, 33261_AT, 40370_F_AT, 37039_AT, 36878_F_AT, 37344_AT	62	42	5085	15.6221	4.88E-05	1.22E-05	5.42E-04
hsa05320:Autoimmun e thyroid disease	8	7.2727	1.93E-06	36773_F_AT, 41723_S_AT, 37421_F_AT, 38095_I_AT, 40369_F_AT, 33261_AT, 40370_F_AT, 37039_AT, 36878_F_AT, 37344_AT	62	51	5085	12.8653	1.93E-04	3.85E-05	0.0021
hsa04612:Antigen processing and presentation	9	8.1818	5.23E-06	36773_F_AT, 41723_S_AT, 35016_AT, 37421_F_AT, 38095_I_AT, 40369_F_AT, 33261_AT, 40370_F_AT, 37039_AT, 36878_F_AT, 37344_AT	62	83	5085	8.8933	5.23E-04	8.71E-05	0.0058
hsa05310:Asthma	6	5.4545	2.00E-05	36773_F_AT, 41723_S_AT, 38095_I_AT, 33261_AT, 37039_AT, 36878_F_AT, 37344_AT	62	29	5085	16.9689	0.0020	2.86E-04	0.0222
hsa04514:Cell adhesion molecules (CAMs)	9	8.1818	1.54E-04	36773_F_AT, 41723_S_AT, 37421_F_AT, 38095_I_AT, 40369_F_AT, 33261_AT, 40370_F_AT, 37039_AT, 36878_F_AT, 37344_AT, 32640_AT	62	132	5085	5.5920	0.0153	0.0019	0.1711
hsa05322:Systemic lupus erythematosus	8	7.2727	1.58E-04	36773_F_AT, 41723_S_AT, 38095_I_AT, 33261_AT, 37039_AT, 36878_F_AT, 37344_AT, 33374_AT, 40766_AT	62	99	5085	6.6276	0.0157	0.0018	0.1754
hsa04672:Intestinal immune network for IgA production	6	5.4545	2.68E-04	36773_F_AT, 41723_S_AT, 38095_I_AT, 33261_AT, 37039_AT, 36878_F_AT, 37344_AT	62	49	5085	10.0428	0.0264	0.0027	0.2966
hsa00590:Arachidonic acid metabolism	6	5.4545	5.03E-04	33777_AT, 37033_S_AT, 38407_R_AT, 1371_S_AT, 32893_S_AT, 216_AT, 715_S_AT, 38081_AT	62	56	5085	8.7874	0.0490	0.0046	0.5564
hsa04640:Hematopoiet ic cell lineage	7	6.3636	5.18E-04	1368_AT, 41723_S_AT, 404_AT, 884_AT, 33261_AT, 39695_AT, 37039_AT	62	86	5085	6.6757	0.0505	0.0043	0.5731
hsa04142:Lysosome	7	6.3636	0.0026	33390_AT, 497_AT, 37021_AT, 37003_AT, 239_AT, 461_AT, 36028_AT	62	117	5085	4.9069	0.2279	0.0197	2.8294
hsa04210:Apoptosis	5	4.5455	0.0199	1368_AT, 1868_G_AT, 1867_AT, 1461_AT, 34892_AT, 1563_S_AT	62	87	5085	4.7136	0.8660	0.1338	19.9982
hsa04610:Complement and coagulation cascades	4	3.6364	0.0492	39695_AT, 36543_AT, 33374_AT, 40766_AT	62	69	5085	4.7546	0.9936	0.2858	42.8973
Adeno-SQ List											
hsa03010:Ribosome	7	14.285 7	4.68E-06	31527_AT, 33656_AT, 32436_AT, 31545_AT, 31505_AT, 33676_AT, 39830_AT	28	87	5085	14.6121	0.0002	0.0002	0.0042
hsa04512:ECM-recept or interaction	6	12.244 9	6.62E-05	34342_S_AT, 2092_S_AT, 37892_AT, 38127_AT, 36929_AT, 406_AT, 33454_AT	28	84	5085	12.9719	0.0025	0.0013	0.0598
hsa04510:Focal adhesion	4	8.1633	0.0888	34342_S_AT, 2092_S_AT, 37892_AT, 36929_AT, 406_AT	28	201	5085	3.6141	0.9708	0.6922	56.8582
Normal-SCLC List											
hsa04510:Focal adhesion	3	10.714 3	0.0286	40480_S_AT, 37671_AT, 36659_AT	8	201	5085	9.4869	0.4567	0.4567	20.0947
Normal-COID List											
hsa04142:Lysosome	5	3.9683	3.09E-02	38597_F_AT, 37176_AT, 39758_F_AT, 38686_AT, 1546_AT, 37105_AT	53	117	5085	4.1001	9.26E-01	9.26E-01	2.85E+01

SCLC-SQ List												
hsa03040:Spliceosome	12	12.5000	1.83E-08	37717_AT, 38455_AT, 40842_AT, 140_S_AT, 35916_S_AT, 41403_AT, 38456_S_AT, 723_S_AT, 36112_R_AT, 32408_S_AT, 37337_AT, 35270_AT, 351_F_AT, 38679_G_AT	50	126	5085	9.6857	9.15E-07	9.15E-07	1.76E-05	
hsa03030:DNA replication	8	8.3333	3.34E-08	1824_S_AT, 947_AT, 40117_AT, 1515_AT, 35312_AT, 38702_AT, 1055_G_AT, 981_AT, 41583_AT, 1884_S_AT	50	36	5085	22.6000	1.67E-06	8.36E-07	3.22E-05	
hsa04110:Cell cycle	10	10.4167	2.20E-06	1824_S_AT, 37458_AT, 947_AT, 40117_AT, 1250_AT, 35312_AT, 1942_S_AT, 36909_AT, 981_AT, 1884_S_AT, 37228_AT	50	125	5085	8.1360	1.10E-04	3.67E-05	0.0021	
hsa03410:Base excision repair	6	6.2500	1.76E-05	1824_S_AT, 32220_AT, 1515_AT, 37686_S_AT, 41146_AT, 38702_AT, 41583_AT, 1287_AT, 1884_S_AT	50	35	5085	17.4343	8.81E-04	2.20E-04	0.0170	
hsa00670:One carbon pool by folate	3	3.1250	0.0100	40074_AT, 674_G_AT, 1505_AT	50	16	5085	19.0687	0.3955	0.0958	9.2400	
