# Supplementary material for: Identification of Single- and Multiple-Class Specific Signature Genes from Gene Expression Profiles by Group Marker Index
Source: PLoS One. 2011 Sep 1;6(9):e24259. doi: 10.1371/journal.pone.0024259 (PMC3164723; doi:10.1371/journal.pone.0024259)
Supplement: Table S11 — The full summary table of the identified pathways related to the level 2 discriminatory genes in the Lung cancer data set (Part II). (PDF) [file pone.0024259.s017.pdf]

Table S11. The full summary table of the identified pathways related to the level 2 discriminatory genes in Lung Cancer data set (Part II).

Term	Count	%	PValue	Genes	List Total	Pop Hits	Pop Total	Fold Enrichment	Bonferroni	Benjamini	FDR
SCLC-COID List											
hsa04020:Calcium signaling pathway	55	2.0992	6.44E-06	40562_AT, 36157_AT, 32691_S_AT, 33954_AT, 36215_AT, 1082_AT, 39125_AT, 33551_S_AT, 34847_S_AT, 2083_AT, 39790_AT, 37450_R_AT, 32294_G_AT, 37134_F_AT, 1158_S_AT, 34846_AT, 36728_AT, 40273_AT, 38236_AT, 33353_AT, 32418_AT, 39124_R_AT, 39123_S_AT, 33626_AT, 33465_AT, 34015_AT, 38580_AT, 35471_G_AT, 336_AT, 41476_AT, 34634_S_AT, 36482_S_AT, 32540_AT, 32419_AT, 901_G_AT, 1336_S_AT, 32907_AT, 1422_G_AT, 38263_AT, 34467_G_AT, 32983_AT, 34466_AT, 438_AT, 32293_AT, 36716_AT, 31550_AT, 35018_AT, 37529_AT, 39275_AT, 37449_I_AT, 36480_AT, 32541_AT, 32688_AT, 32998_AT, 37273_AT, 41028_AT, 37448_S_AT, 40330_AT, 31811_R_AT, 41144_G_AT, 900_AT, 564_AT, 281_S_AT, 41052_S_AT, 32689_S_AT, 363_AT, 1419_G_AT, 37053_AT, 38001_AT, 32456_S_AT	880	176	5085	1.8058	1.23E-03	1.23E-03	0.0080
hsa05223:Non-small cell lung cancer	24	0.9160	1.06E-05	37961_AT, 303_AT, 1336_S_AT, 322_AT, 1082_AT, 40972_AT, 35373_AT, 1844_S_AT, 37763_AT, 1992_AT, 1900_AT, 1381_AT, 1207_AT, 1861_AT, 36294_AT, 1000_AT, 1974_S_AT, 1570_F_AT, 1322_AT, 32029_AT, 1048_AT, 34197_AT, 34479_AT, 1130_AT, 41546_AT, 1707_G_AT, 1362_S_AT, 40704_AT, 363_AT, 2028_S_AT, 31699_AT	880	54	5085	2.5682	0.0020	1.01E-03	0.0132
hsa03040:Spliceosome	40	1.5267	1.01E-04	33631_AT, 34149_AT, 41746_AT, 34286_AT, 34365_AT, 38654_AT, 41712_AT, 39415_AT, 38678_AT, 39343_AT, 40820_AT, 38680_AT, 32125_AT, 37379_AT, 32753_AT, 40656_AT, 32124_AT, 32165_AT, 37949_AT, 34270_AT, 37715_AT, 40036_AT, 35809_G_AT, 1621_AT, 34647_AT, 32556_AT, 40875_S_AT, 40269_AT, 32557_AT, 33817_AT, 40453_S_AT, 35286_R_AT, 35306_AT, 40819_AT, 40457_AT, 40904_AT, 38040_AT, 40905_S_AT, 35808_AT, 32560_S_AT, 33237_AT, 37935_AT, 39426_AT, 41370_AT, 32559_S_AT, 34366_G_AT, 37390_AT, 38030_AT	880	126	5085	1.8344	0.0191	0.0064	0.1256
hsa04070:Phosphatidylinositol signaling system	27	1.0305	1.54E-04	901_G_AT, 37961_AT, 1336_S_AT, 39323_AT, 322_AT, 33954_AT, 31833_AT, 1082_AT, 35373_AT, 41692_AT, 38003_S_AT, 41227_AT, 1434_AT, 34169_S_AT, 36532_AT, 40253_AT, 35665_AT, 33397_AT, 1158_S_AT, 40783_S_AT, 41343_AT, 40330_AT, 41144_G_AT, 36598_S_AT, 32697_AT, 34197_AT, 34479_AT, 39552_AT, 900_AT, 31400_AT, 363_AT, 40704_AT, 31699_AT	880	74	5085	2.1083	0.0289	0.0073	0.1909
hsa04722:Neurotrophin signaling pathway	39	1.4885	1.56E-04	37961_AT, 303_AT, 33009_AT, 37839_AT, 1082_AT, 40972_AT, 1355_G_AT, 34847_S_AT, 1060_G_AT, 1708_AT, 41444_AT, 2065_S_AT, 35041_AT, 1010_AT, 1158_S_AT, 34846_AT, 41432_AT, 1974_S_AT, 1000_AT, 40033_AT, 34006_S_AT, 36805_S_AT, 409_AT, 1354_AT, 34197_AT, 1059_AT, 40704_AT, 38443_AT, 2060_AT, 1636_G_AT, 322_AT, 1422_G_AT, 1709_G_AT, 35373_AT, 1844_S_AT, 1858_AT, 791_G_AT, 1861_AT, 1671_S_AT, 2041_I_AT, 2071_S_AT, 40645_AT, 40149_AT, 41144_G_AT,	880	124	5085	1.8174	0.0294	0.0059	0.1941

				35622_AT, 34479_AT, 1130_AT, 1088_AT, 1698_G_AT, 39163_AT, 32530_AT, 31699_AT, 40023_AT							
hsa04210:Apoptosis	30	1.1450	1.89E-04	37961_AT, 33056_AT, 40696_AT, 322_AT, 36215_AT, 40972_AT, 35373_AT, 1646_AT, 1858_AT, 116_AT, 33057_G_AT, 438_AT, 1765_AT, 2065_S_AT, 32726_G_AT, 791_G_AT, 1861_AT, 35018_AT, 32541_AT, 35604_AT, 35537_AT, 1974_S_AT, 36805_S_AT, 36004_AT, 34479_AT, 34197_AT, 1076_AT, 281_S_AT, 34493_AT, 475_AT, 40704_AT, 37227_AT, 2060_AT, 31699_AT, 37221_AT, 32540_AT	880	87	5085	1.9926	0.0355	0.0060	0.2350
hsa05214:Glioma	24	0.9160	1.90E-04	37961_AT, 303_AT, 1336_S_AT, 1880_AT, 36157_AT, 322_AT, 1082_AT, 40972_AT, 34847_S_AT, 35373_AT, 1844_S_AT, 1859_S_AT, 2008_S_AT, 1900_AT, 1434_AT, 1207_AT, 1158_S_AT, 34846_AT, 1000_AT, 1974_S_AT, 1570_F_AT, 1322_AT, 40139_AT, 41144_G_AT, 35622_AT, 34197_AT, 34479_AT, 41546_AT, 1130_AT, 39552_AT, 1707_G_AT, 40704_AT, 363_AT, 2028_S_AT, 31699_AT	880	63	5085	2.2013	0.0357	0.0052	0.2363
hsa04010:MAPK signaling pathway	70	2.6718	1.95E-04	303_AT, 33009_AT, 36157_AT, 40363_R_AT, 39647_S_AT, 36215_AT, 40972_AT, 1355_G_AT, 36168_AT, 37711_AT, 35652_G_AT, 36764_AT, 310_S_AT, 36905_AT, 1708_AT, 41444_AT, 392_G_AT, 41279_F_AT, 35041_AT, 38289_R_AT, 35081_AT, 40656_AT, 41280_R_AT, 1010_AT, 32665_AT, 1781_AT, 41432_AT, 36294_AT, 1974_S_AT, 331_AT, 1000_AT, 40033_AT, 34006_S_AT, 36805_S_AT, 36004_AT, 1354_AT, 33626_AT, 35651_AT, 41226_AT, 1076_AT, 37588_S_AT, 36230_AT, 1380_AT, 35328_AT, 35527_AT, 1994_AT, 32540_AT, 41674_AT, 1336_S_AT, 41645_AT, 1732_AT, 1616_AT, 39965_AT, 1709_G_AT, 34415_AT, 1844_S_AT, 1858_AT, 41587_G_AT, 33275_AT, 35634_AT, 438_AT, 41586_AT, 791_G_AT, 41445_AT, 35018_AT, 37529_AT, 1671_S_AT, 39275_AT, 32541_AT, 37575_AT, 1593_AT, 2071_S_AT, 38902_R_AT, 36435_AT, 1981_S_AT, 39199_AT, 1130_AT, 1088_AT, 41588_AT, 1562_G_AT, 281_S_AT, 857_AT, 391_AT, 1698_G_AT, 38629_AT, 41052_S_AT, 363_AT, 38001_AT, 36935_AT, 1782_S_AT, 113_I_AT, 40023_AT	880	267	5085	1.5149	0.0366	0.0047	0.2428
hsa05218:Melanoma	26	0.9924	1.97E-04	37961_AT, 41674_AT, 36157_AT, 1880_AT, 322_AT, 1732_AT, 1616_AT, 40972_AT, 1095_S_AT, 1844_S_AT, 35373_AT, 36168_AT, 1859_S_AT, 41587_G_AT, 2008_S_AT, 41586_AT, 1900_AT, 1434_AT, 1207_AT, 35081_AT, 1861_AT, 1608_AT, 1974_S_AT, 1000_AT, 1570_F_AT, 1322_AT, 1593_AT, 34197_AT, 34479_AT, 41546_AT, 1130_AT, 39552_AT, 41588_AT, 1707_G_AT, 1380_AT, 40704_AT, 2028_S_AT, 31699_AT	880	71	5085	2.1160	0.0369	0.0042	0.2448
hsa05220:Chronic myeloid leukemia	27	1.0305	1.97E-04	37961_AT, 303_AT, 1880_AT, 322_AT, 40972_AT, 1844_S_AT, 34415_AT, 35373_AT, 1859_S_AT, 2008_S_AT, 509_AT, 1900_AT, 1207_AT, 41445_AT, 1861_AT, 31443_AT, 510_G_AT, 1339_S_AT, 2041_I_AT, 1974_S_AT, 1000_AT, 1570_F_AT, 1322_AT, 35622_AT, 36004_AT, 34197_AT, 34479_AT, 41546_AT, 1130_AT,	880	75	5085	2.0802	0.0370	0.0038	0.2452

				39199_AT, 40458_AT, 1707_G_AT, 472_AT, 40704_AT, 2028_S_AT, 38443_AT, 1636_G_AT, 31699_AT, 780_AT								
hsa04110:Cell cycle	38	1.4504	4.09E-04	40921_AT, 1880_AT, 37758_S_AT, 33831_AT, 31877_AT, 1859_S_AT, 41532_AT, 1900_AT, 1584_AT, 32596_AT, 41623_S_AT, 1974_S_AT, 1570_F_AT, 1797_AT, 1322_AT, 409_AT, 40590_AT, 41546_AT, 34709_R_AT, 480_AT, 41497_AT, 34741_AT, 1636_G_AT, 38564_AT, 40404_S_AT, 38155_AT, 39724_S_AT, 35835_AT, 35546_AT, 36895_AT, 37238_S_AT, 509_AT, 2008_S_AT, 39855_AT, 39723_AT, 2013_AT, 1207_AT, 38920_AT, 41445_AT, 510_G_AT, 35578_AT, 2041_I_AT, 40645_AT, 1924_AT, 31631_F_AT, 34763_AT, 32530_AT, 2028_S_AT, 36053_AT, 37282_AT, 38158_AT, 39246_AT	880	125	5085	1.7566	0.0752	0.0071	0.5081
hsa04012:ErbB signaling pathway	29	1.1069	4.68E-04	37961_AT, 303_AT, 1336_S_AT, 38159_AT, 322_AT, 1709_G_AT, 1082_AT, 40972_AT, 1844_S_AT, 35373_AT, 34847_S_AT, 33275_AT, 33075_AT, 1708_AT, 41444_AT, 35091_AT, 1861_AT, 1781_AT, 34846_AT, 2041_I_AT, 1000_AT, 37273_AT, 34006_S_AT, 2071_S_AT, 40645_AT, 40139_AT, 35622_AT, 34197_AT, 34479_AT, 1130_AT, 40458_AT, 39213_AT, 1707_G_AT, 472_AT, 363_AT, 40704_AT, 1636_G_AT, 31699_AT	880	87	5085	1.9261	0.0855	0.0074	0.5806
hsa04930:Type II diabetes mellitus	19	0.7252	4.79E-04	37961_AT, 40925_AT, 322_AT, 41793_AT, 40969_AT, 1709_G_AT, 35373_AT, 1708_AT, 38238_AT, 33162_AT, 39275_AT, 1000_AT, 34006_S_AT, 40658_R_AT, 2071_S_AT, 40139_AT, 34479_AT, 33626_AT, 34197_AT, 40657_R_AT, 41052_S_AT, 40704_AT, 38001_AT, 41792_AT, 1572_S_AT, 31699_AT	880	47	5085	2.3360	0.0874	0.0070	0.5937
hsa05210:Colorectal cancer	28	1.0687	6.01E-04	37961_AT, 31440_AT, 303_AT, 36157_AT, 322_AT, 35433_S_AT, 39965_AT, 1709_G_AT, 40972_AT, 1844_S_AT, 1017_AT, 34415_AT, 35373_AT, 509_AT, 1708_AT, 2065_S_AT, 41445_AT, 1861_AT, 1608_AT, 510_G_AT, 1912_S_AT, 1974_S_AT, 1000_AT, 34006_S_AT, 2071_S_AT, 40645_AT, 34197_AT, 34479_AT, 1130_AT, 39199_AT, 1719_AT, 1707_G_AT, 40704_AT, 2060_AT, 31699_AT, 34184_AT	880	84	5085	1.9261	0.1085	0.0082	0.7453
hsa05213:Endometrial cancer	20	0.7634	6.70E-04	37961_AT, 31440_AT, 303_AT, 322_AT, 35433_S_AT, 40972_AT, 35373_AT, 1844_S_AT, 33275_AT, 1434_AT, 1861_AT, 1781_AT, 1912_S_AT, 1000_AT, 1974_S_AT, 32029_AT, 40645_AT, 34197_AT, 34479_AT, 1130_AT, 39552_AT, 1707_G_AT, 31841_AT, 40704_AT, 31699_AT, 34184_AT	880	52	5085	2.2225	0.1201	0.0085	0.8297
hsa05200:Pathways in cancer	80	3.0534	7.56E-04	35352_AT, 37961_AT, 303_AT, 1880_AT, 36157_AT, 40507_AT, 40363_R_AT, 34456_S_AT, 421_AT, 40972_AT, 36168_AT, 1859_S_AT, 1708_AT, 1900_AT, 1381_AT, 35081_AT, 1974_S_AT, 35638_AT, 34006_S_AT, 1322_AT, 37268_AT, 36805_S_AT, 36004_AT, 34197_AT, 41546_AT, 39552_AT, 40643_AT, 40458_AT, 1362_S_AT, 31841_AT, 40704_AT, 2060_AT, 780_AT, 1636_G_AT, 31440_AT, 1336_S_AT, 322_AT, 35641_G_AT, 39965_AT, 1709_G_AT, 41587_G_AT, 509_AT, 41586_AT, 32726_G_AT, 1018_AT, 1207_AT, 510_G_AT, 1339_S_AT,	880	328	5085	1.4094	0.1345	0.0090	0.9364

				2041_I_AT, 37144_AT, 2071_S_AT, 40645_AT, 1981_S_AT, 39199_AT, 41588_AT, 363_AT, 1419_G_AT, 35433_S_AT, 33831_AT, 1082_AT, 1095_S_AT, 1646_AT, 37433_AT, 2065_S_AT, 1434_AT, 37432_G_AT, 37431_AT, 1608_AT, 35640_AT, 36294_AT, 1000_AT, 1570_F_AT, 1048_AT, 40139_AT, 1503_AT, 1707_G_AT, 472_AT, 1380_AT, 41674_AT, 33684_AT, 1732_AT, 1616_AT, 1017_AT, 34415_AT, 35373_AT, 1844_S_AT, 1858_AT, 37763_AT, 2008_S_AT, 37057_S_AT, 41445_AT, 1861_AT, 31443_AT, 1912_S_AT, 1019_G_AT, 33986_R_AT, 1593_AT, 34479_AT, 1130_AT, 1926_AT, 1719_AT, 121_AT, 2028_S_AT, 31699_AT, 36573_AT, 34184_AT							
hsa05010:Alzheimer's disease	45	1.7176	0.0011	38695_AT, 35264_AT, 799_AT, 38777_AT, 33954_AT, 310_S_AT, 39790_AT, 40565_AT, 40096_AT, 32752_AT, 37134_F_AT, 1158_S_AT, 331_AT, 1000_AT, 38236_AT, 38605_AT, 38580_AT, 41760_AT, 38660_AT, 34381_AT, 36482_S_AT, 37227_AT, 32540_AT, 35364_AT, 38979_AT, 38451_AT, 901_G_AT, 38983_AT, 1101_AT, 38080_AT, 36107_AT, 32726_G_AT, 800_G_AT, 35018_AT, 1861_AT, 39275_AT, 35774_R_AT, 36165_AT, 32541_AT, 40549_AT, 41028_AT, 40645_AT, 41144_G_AT, 38462_AT, 900_AT, 281_S_AT, 38629_AT, 38001_AT, 39427_AT, 113_I_AT, 41206_R_AT, 39482_AT	880	163	5085	1.5953	0.1875	0.0121	1.3434
hsa05215:Prostate cancer	28	1.0687	0.0016	37961_AT, 31440_AT, 303_AT, 36157_AT, 1880_AT, 322_AT, 37535_AT, 33831_AT, 40972_AT, 1844_S_AT, 35373_AT, 36168_AT, 1859_S_AT, 2008_S_AT, 1900_AT, 1434_AT, 1861_AT, 1974_S_AT, 1000_AT, 1570_F_AT, 1322_AT, 33986_R_AT, 32029_AT, 877_AT, 40849_S_AT, 40645_AT, 40139_AT, 36004_AT, 34197_AT, 34479_AT, 1130_AT, 39552_AT, 1707_G_AT, 40704_AT, 2028_S_AT, 2060_AT, 31699_AT	880	89	5085	1.8179	0.2646	0.0169	1.9818
hsa04720:Long-term potentiation	23	0.8779	0.0017	901_G_AT, 37725_AT, 1336_S_AT, 40438_AT, 1422_G_AT, 36215_AT, 33954_AT, 33831_AT, 34847_S_AT, 1844_S_AT, 438_AT, 35018_AT, 37134_F_AT, 1158_S_AT, 32541_AT, 34846_AT, 1000_AT, 33353_AT, 38236_AT, 41144_G_AT, 1130_AT, 38580_AT, 900_AT, 1707_G_AT, 281_S_AT, 363_AT, 38001_AT, 32540_AT	880	68	5085	1.9545	0.2790	0.0171	2.1081
hsa04914:Progesterone -mediated oocyte maturation	27	1.0305	0.0021	37961_AT, 35835_AT, 322_AT, 746_AT, 36215_AT, 1709_G_AT, 31877_AT, 40972_AT, 1844_S_AT, 35373_AT, 37238_S_AT, 1708_AT, 39855_AT, 438_AT, 1584_AT, 1671_S_AT, 1010_AT, 41623_S_AT, 1000_AT, 40033_AT, 34006_S_AT, 33353_AT, 33986_R_AT, 2071_S_AT, 40590_AT, 876_AT, 34197_AT, 34479_AT, 1130_AT, 480_AT, 1707_G_AT, 40704_AT, 37282_AT, 31699_AT, 40404_S_AT	880	86	5085	1.8142	0.3252	0.0195	2.5289
hsa04310:Wnt signaling pathway	41	1.5649	0.0026	176_AT, 36215_AT, 35433_S_AT, 38019_AT, 33831_AT, 34697_AT, 31365_F_AT, 1093_AT, 34847_S_AT, 36905_AT, 1708_AT, 39068_AT, 40786_AT, 594_S_AT, 32271_AT, 31364_I_AT, 36235_AT, 34846_AT, 1974_S_AT, 34006_S_AT, 635_S_AT, 32540_AT, 34735_AT, 901_G_AT, 31440_AT, 39724_S_AT, 33684_AT, 1336_S_AT,	880	151	5085	1.5690	0.3949	0.0236	3.2188

				39965_AT, 1709_G_AT, 509_AT, 40981_AT, 438_AT, 34956_AT, 39723_AT, 1018_AT, 35018_AT, 33753_AT, 32385_AT, 32541_AT, 510_G_AT, 1912_S_AT, 40867_AT, 1019_G_AT, 40980_AT, 2071_S_AT, 40645_AT, 33935_AT, 237_S_AT, 922_AT, 33594_AT, 900_AT, 281_S_AT, 32854_AT, 363_AT, 34184_AT							
hsa04114:Oocyte meiosis	32	1.2214	0.0028	37725_AT, 39724_S_AT, 176_AT, 35835_AT, 33954_AT, 36215_AT, 35546_AT, 1093_AT, 31877_AT, 1844_S_AT, 34847_S_AT, 37238_S_AT, 438_AT, 40786_AT, 39723_AT, 39068_AT, 1584_AT, 35018_AT, 1158_S_AT, 32541_AT, 34846_AT, 1000_AT, 33353_AT, 40867_AT, 409_AT, 41144_G_AT, 40590_AT, 237_S_AT, 1130_AT, 922_AT, 480_AT, 34763_AT, 31631_F_AT, 635_S_AT, 281_S_AT, 32854_AT, 32530_AT, 37282_AT, 32540_AT, 38158_AT, 40404_S_AT	880	110	5085	1.6810	0.4135	0.0240	3.4154
hsa05222:Small cell lung cancer	26	0.9924	0.0031	37961_AT, 322_AT, 40972_AT, 35373_AT, 1646_AT, 37763_AT, 37433_AT, 1992_AT, 37057_S_AT, 1900_AT, 1434_AT, 1207_AT, 1381_AT, 37432_G_AT, 37431_AT, 1974_S_AT, 1570_F_AT, 1322_AT, 1048_AT, 37144_AT, 36004_AT, 34479_AT, 1981_S_AT, 34197_AT, 41546_AT, 39552_AT, 40643_AT, 1362_S_AT, 40704_AT, 1419_G_AT, 2028_S_AT, 37227_AT, 2060_AT, 31699_AT, 36573_AT	880	84	5085	1.7886	0.4524	0.0258	3.8464
hsa04622:RIG-I-like receptor signaling pathway	23	0.8779	0.0032	40696_AT, 33009_AT, 1709_G_AT, 32415_AT, 36905_AT, 1708_AT, 40421_AT, 1765_AT, 37057_S_AT, 39582_AT, 33285_I_AT, 1671_S_AT, 1010_AT, 427_F_AT, 33955_AT, 40033_AT, 34006_S_AT, 2071_S_AT, 1666_AT, 36004_AT, 1540_F_AT, 1022_F_AT, 1770_AT, 826_AT, 39013_AT, 38600_R_AT, 475_AT, 1075_F_AT	880	71	5085	1.8719	0.4533	0.0248	3.8563
hsa05212:Pancreatic cancer	23	0.8779	0.0038	37961_AT, 322_AT, 39965_AT, 1709_G_AT, 40972_AT, 1844_S_AT, 35373_AT, 34415_AT, 509_AT, 1708_AT, 1900_AT, 1207_AT, 41445_AT, 1861_AT, 510_G_AT, 1000_AT, 1974_S_AT, 1570_F_AT, 34006_S_AT, 1322_AT, 37268_AT, 2071_S_AT, 36004_AT, 34197_AT, 34479_AT, 41546_AT, 1130_AT, 39199_AT, 1503_AT, 1926_AT, 1707_G_AT, 40704_AT, 2028_S_AT, 31699_AT	880	72	5085	1.8459	0.5188	0.0288	4.6529
hsa04140:Regulation of autophagy	14	0.5344	0.0039	37901_AT, 1666_AT, 39776_AT, 1540_F_AT, 1022_F_AT, 37957_AT, 32415_AT, 1770_AT, 34827_AT, 39013_AT, 1611_S_AT, 38600_R_AT, 1075_F_AT, 427_F_AT, 35665_AT	880	35	5085	2.3114	0.5298	0.0286	4.7956
