# Supplementary material for: Identification of Single- and Multiple-Class Specific Signature Genes from Gene Expression Profiles by Group Marker Index
Source: PLoS One. 2011 Sep 1;6(9):e24259. doi: 10.1371/journal.pone.0024259 (PMC3164723; doi:10.1371/journal.pone.0024259)
Supplement: File S1 — The level-2 genes selected only by GMI and the level-2 genes selected only by TBM in the Lung Cancer data set. (PDF) [file pone.0024259.s018.pdf]

The level-2 genes selected only by GMI in the Lung Cancer data set:

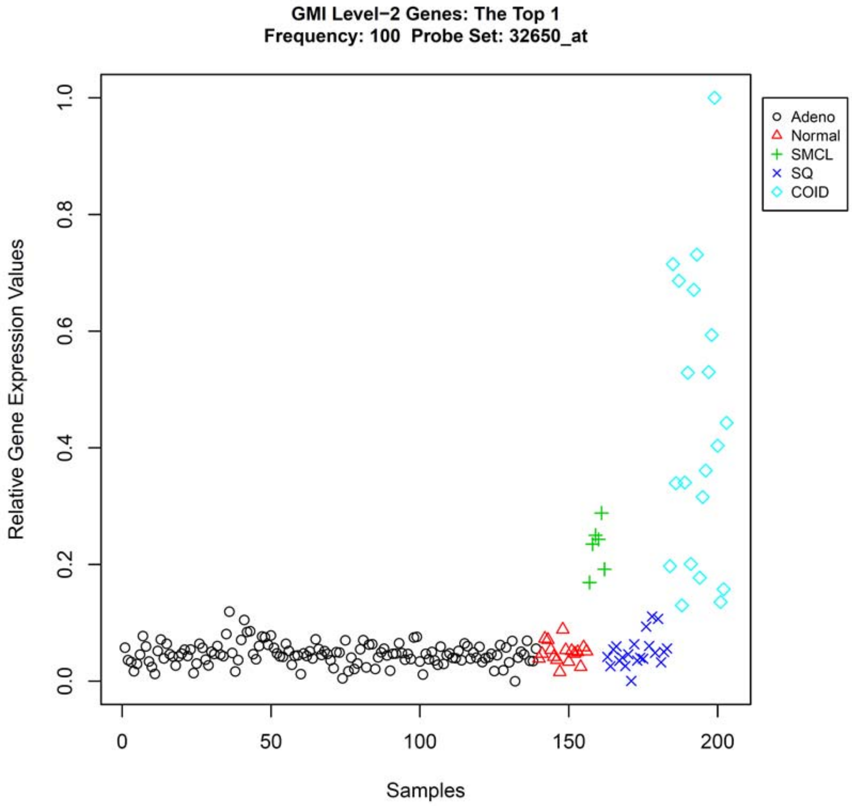

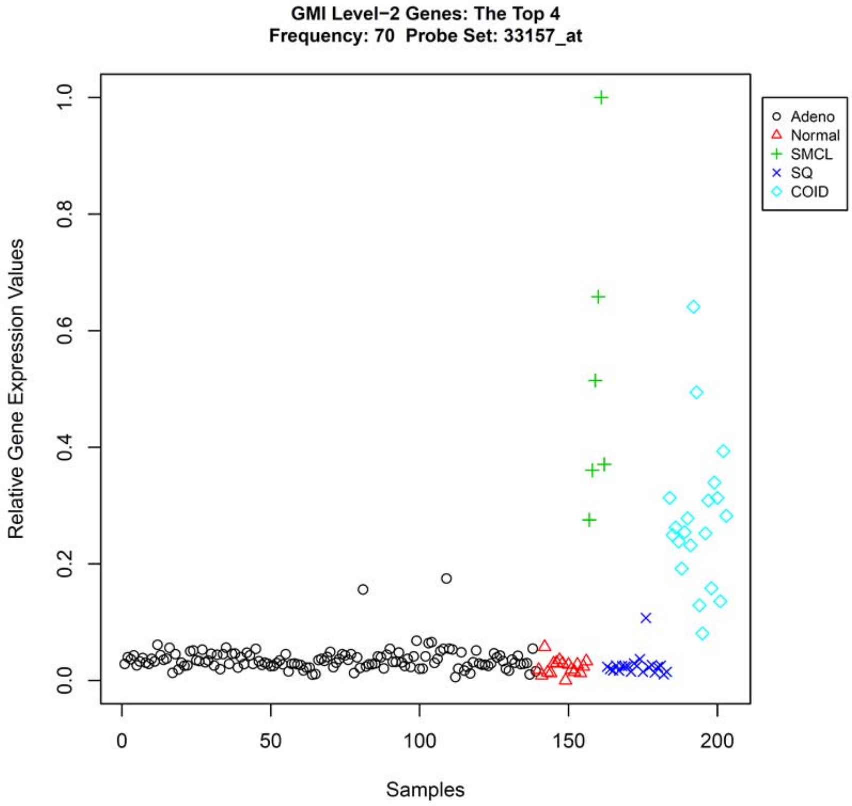

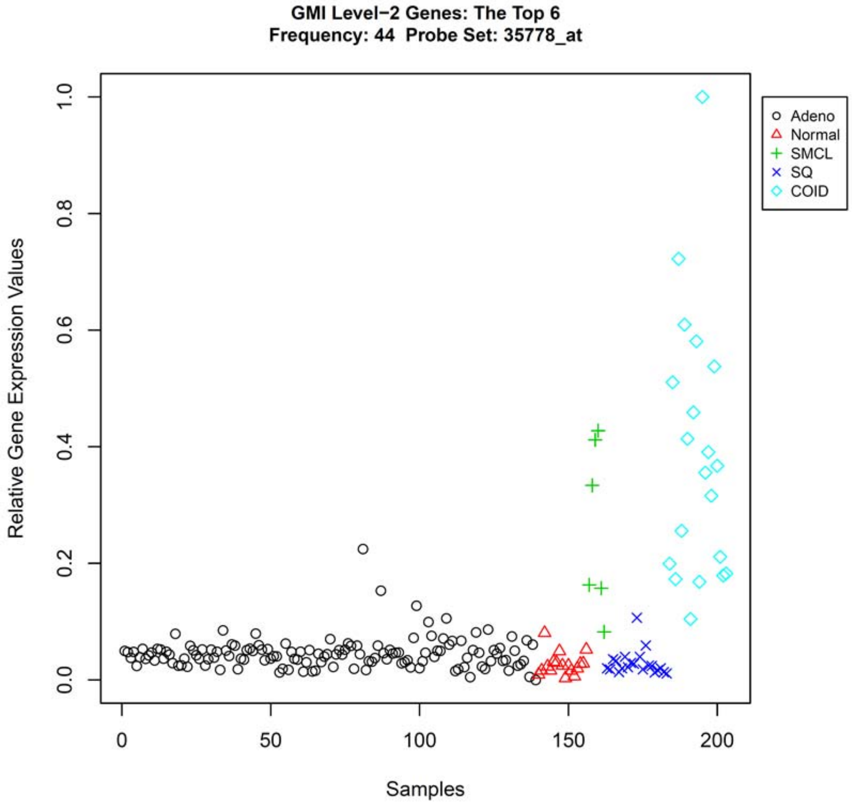
GMI Level−2 Genes: The Top 6
Frequency: 44 Probe Set: 35778_at
Relative Gene Expression Values
Samples
Adeno
Normal
SMCL
SQ
COID

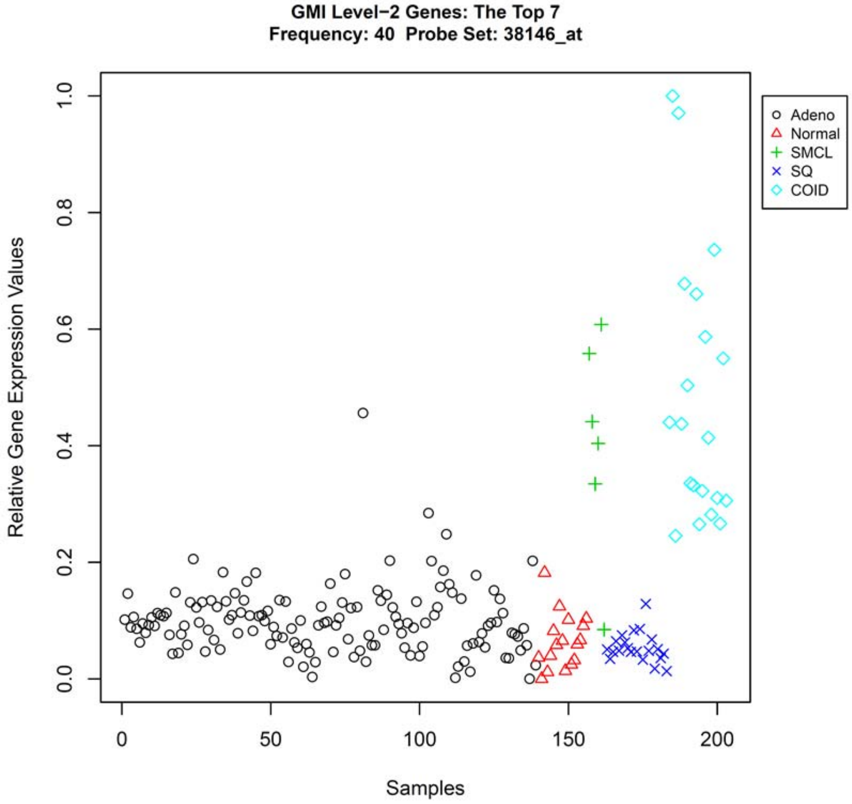
GMI Level−2 Genes: The Top 7
Frequency: 40 Probe Set: 38146_at
Relative Gene Expression Values
Samples
Adeno
Normal
SMCL
SQ
COID

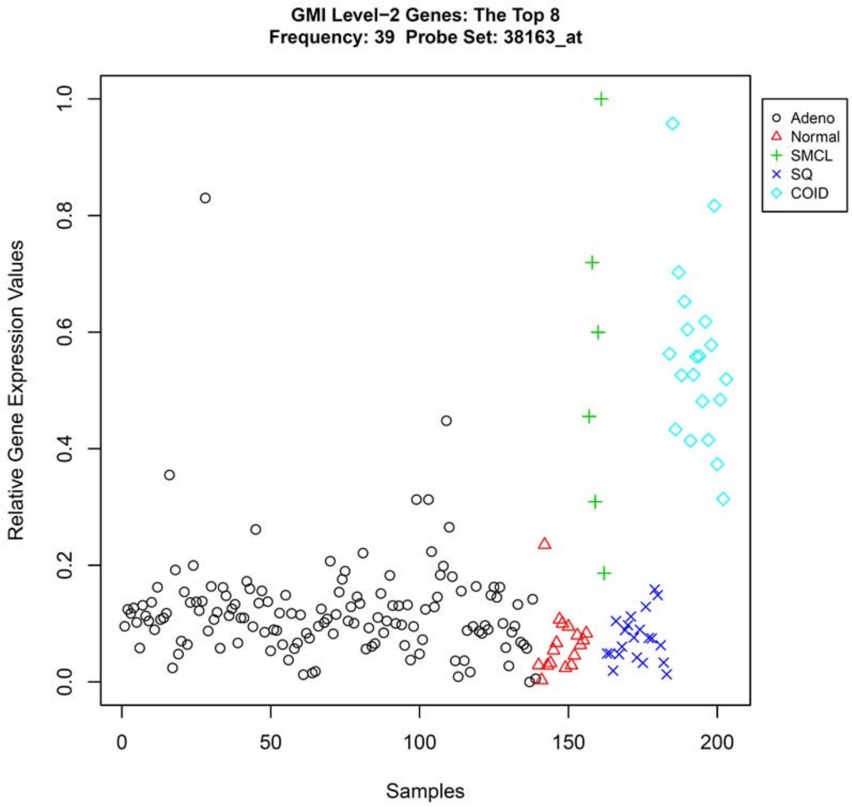
GMI Level−2 Genes: The Top 8
Frequency: 39 Probe Set: 38163_at
Relative Gene Expression Values
Samples
Adeno
Normal
SMCL
SQ
COID
0.0
0.2
0.4
0.6
0.8
1.0
0
50
100
150
200

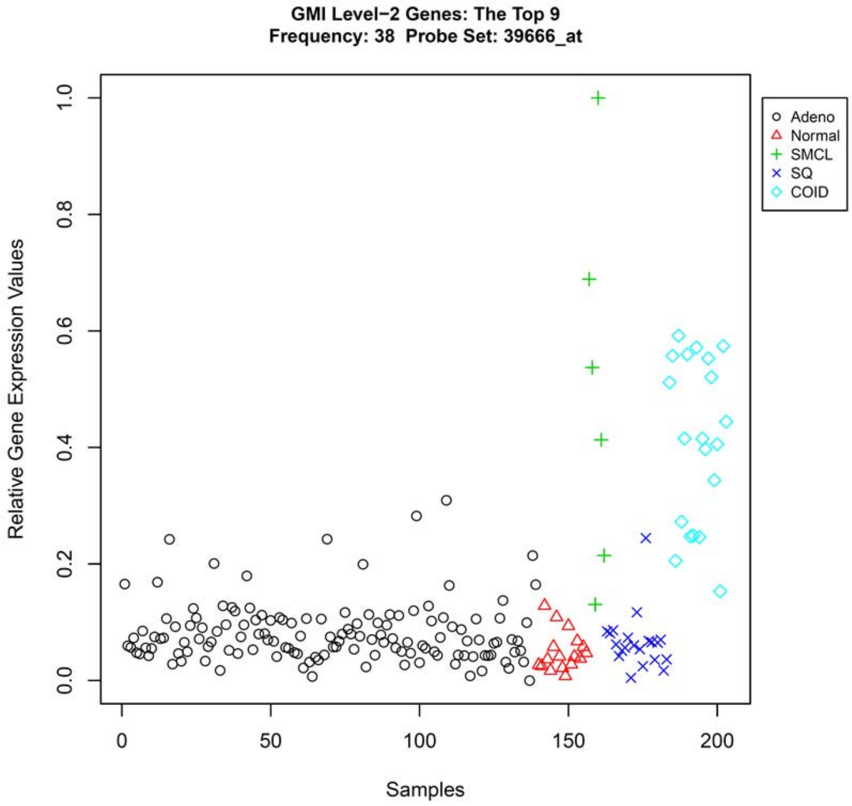
GMI Level−2 Genes: The Top 9
Frequency: 38 Probe Set: 39666_at
Relative Gene Expression Values
Samples
Adeno
Normal
SMCL
SQ
COID
0.0
0.2
0.4
0.6
0.8
1.0
0
50
100
150
200

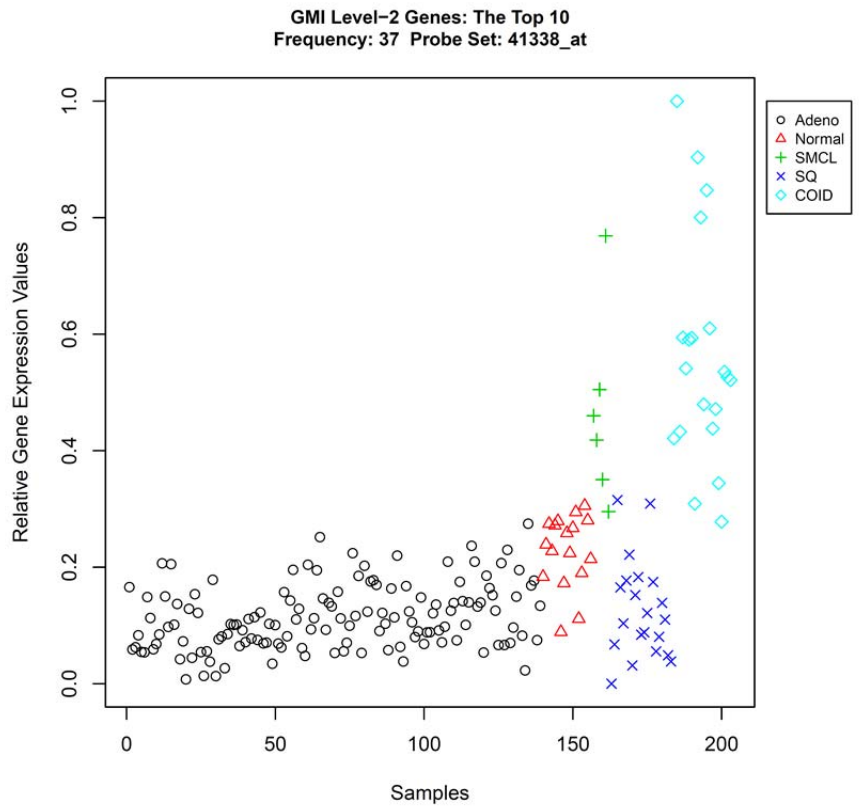

The level-2 genes selected only by TBM in the Lung Cancer data set:

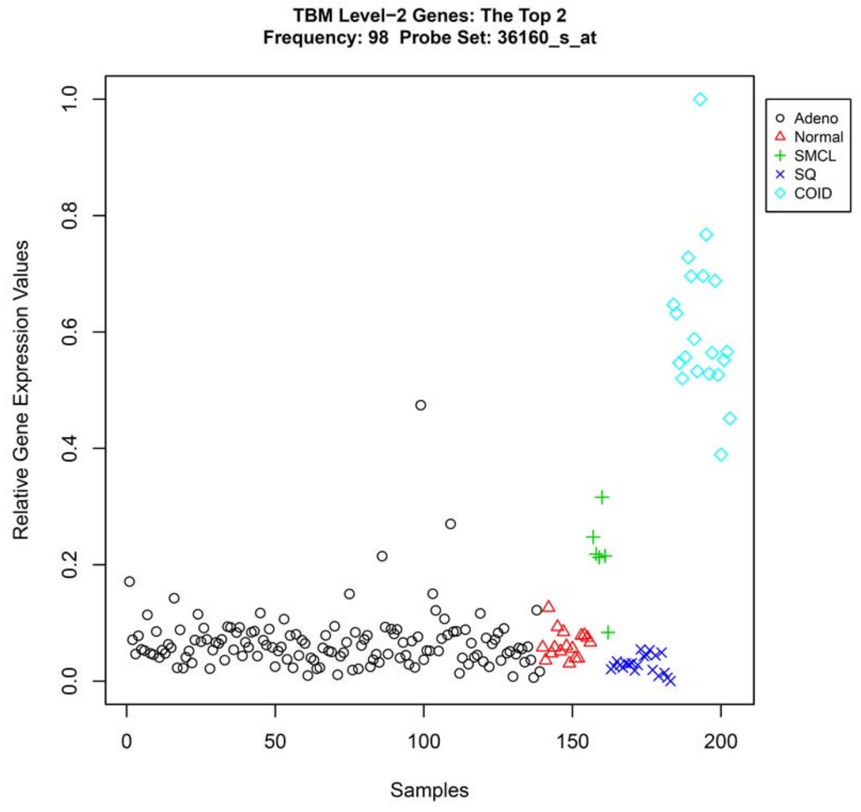

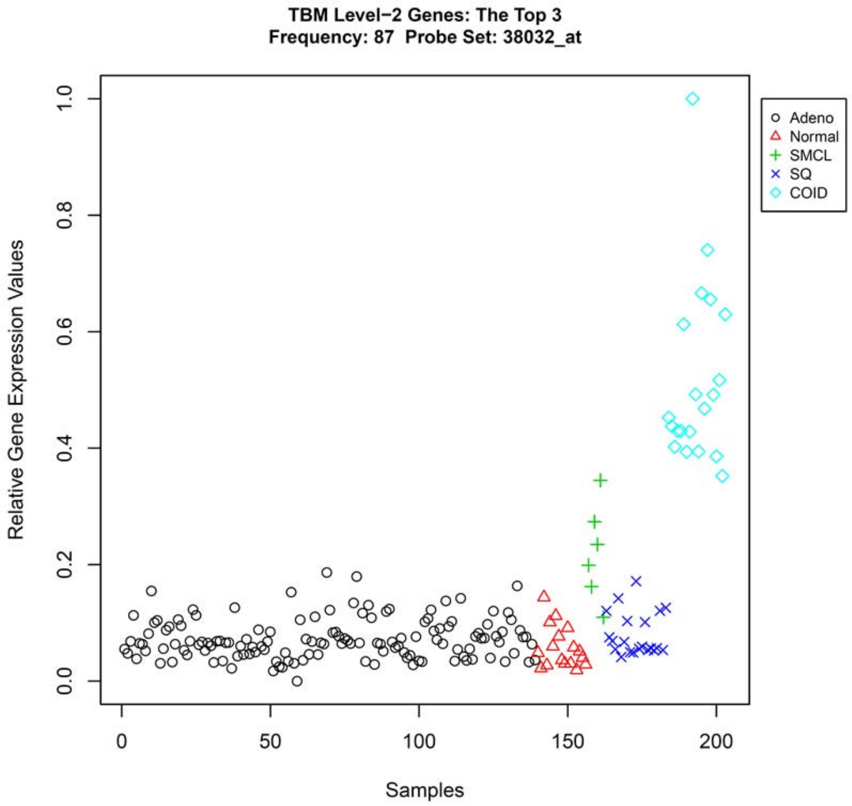
TBM Level−2 Genes: The Top 3
Frequency: 87 Probe Set: 38032_at
Relative Gene Expression Values
Samples
0.0
0.2
0.4
0.6
0.8
1.0
0
50
100
150
200
Adeno
Normal
SMCL
SQ
COID

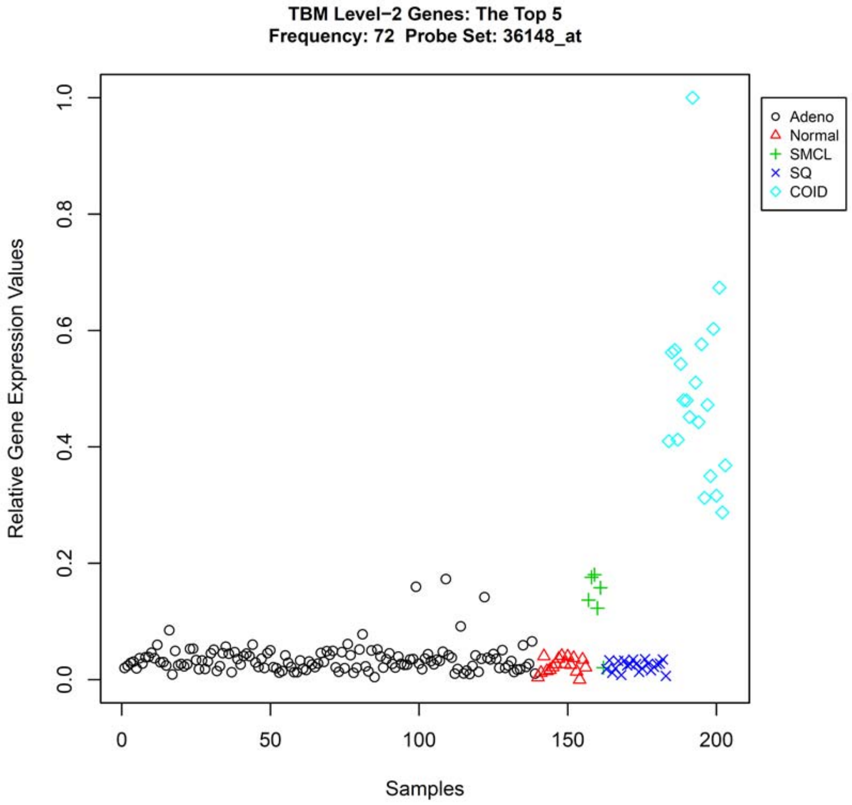
TBM Level−2 Genes: The Top 5
Frequency: 72 Probe Set: 36148_at
Relative Gene Expression Values
Samples
0.0
0.2
0.4
0.6
0.8
1.0
0
50
100
150
200
Adeno
Normal
SMCL
SQ
COID

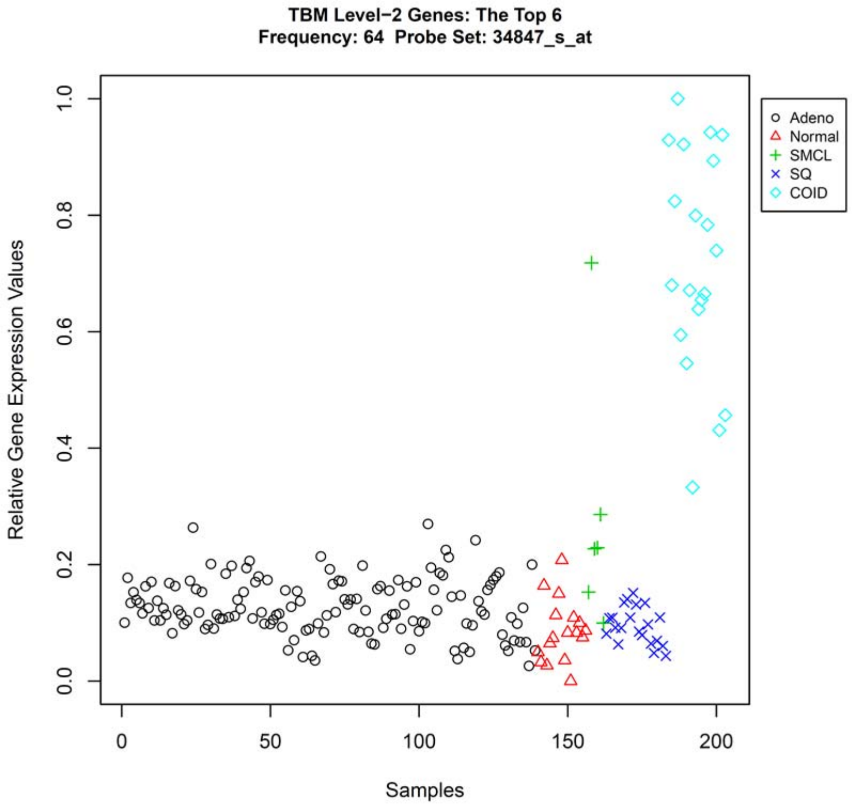
TBM Level−2 Genes: The Top 6
Frequency: 64 Probe Set: 34847_s_at
Relative Gene Expression Values
Samples
Adeno
Normal
SMCL
SQ
COID
0.0
0.2
0.4
0.6
0.8
1.0
0
50
100
150
200

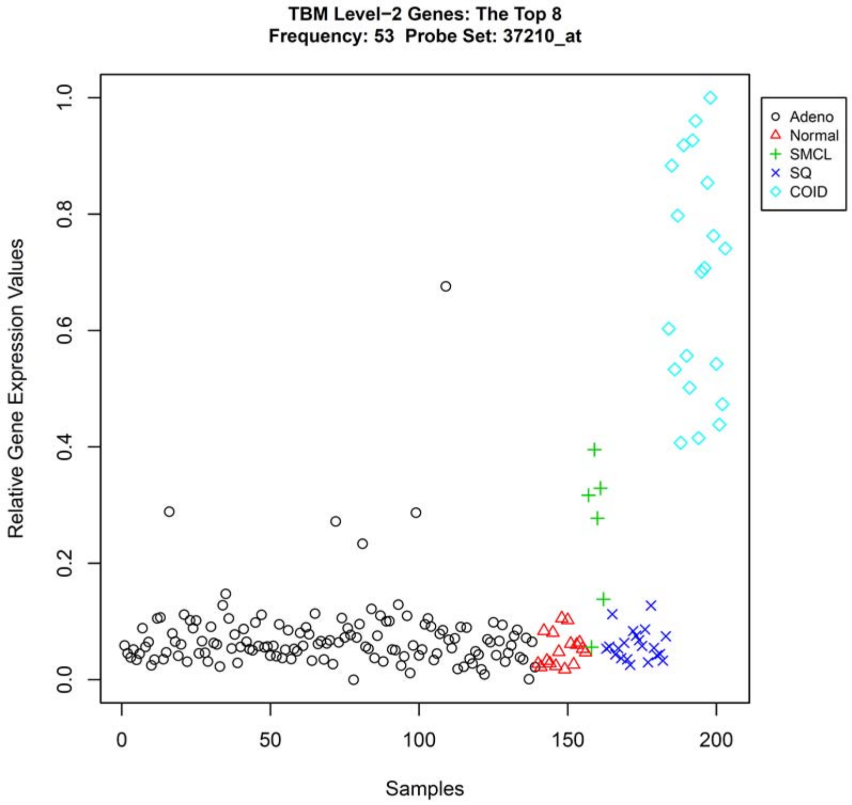
TBM Level−2 Genes: The Top 8
Frequency: 53 Probe Set: 37210_at
Relative Gene Expression Values
Samples
Adeno
Normal
SMCL
SQ
COID
0.0
0.2
0.4
0.6
0.8
1.0
0
50
100
150
200

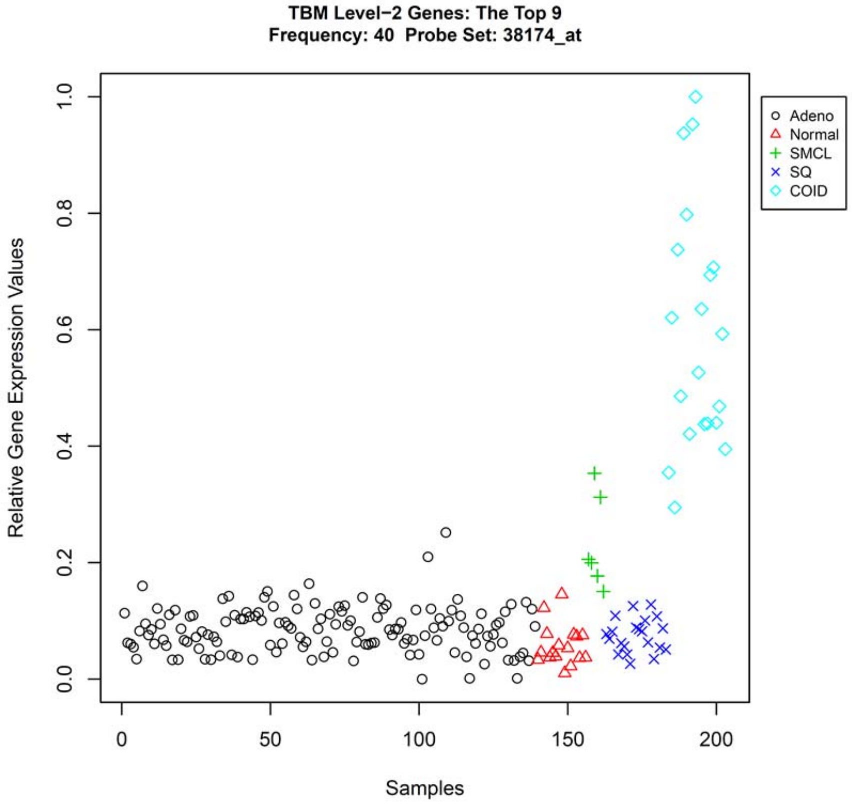
TBM Level−2 Genes: The Top 9
Frequency: 40 Probe Set: 38174_at
Relative Gene Expression Values
Samples
Adeno
Normal
SMCL
SQ
COID

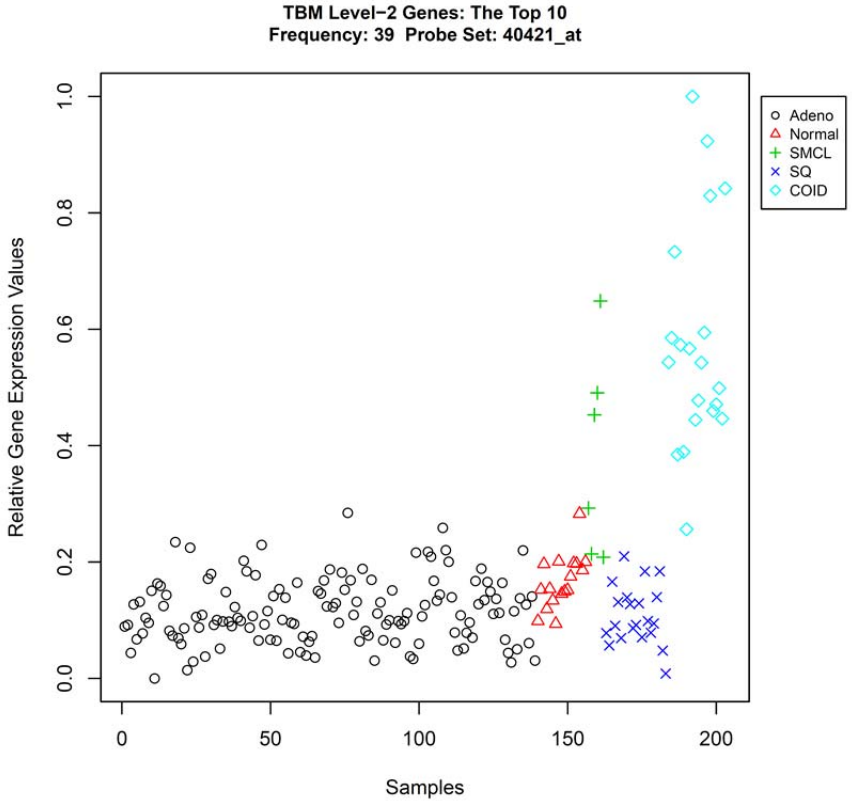
TBM Level−2 Genes: The Top 10
Frequency: 39 Probe Set: 40421_at
Relative Gene Expression Values
Samples
Adeno
Normal
SMCL
SQ
COID
